# On the Meaning of the “*P* Factor” in Symmetrical Bifactor Models of Psychopathology: Recommendations for Future Research From the Bifactor-(*S*−1) Perspective

**DOI:** 10.1177/10731911211060298

**Published:** 2021-12-03

**Authors:** Manuel Heinrich, Christian Geiser, Pavle Zagorscak, G. Leonard Burns, Johannes Bohn, Stephen P. Becker, Michael Eid, Theodore P. Beauchaine, Christine Knaevelsrud

**Affiliations:** 1 Freie Universität Berlin, Germany; 2 Utah State University, Logan, USA; 3 Washington State University, Pullman, USA; 4 University of Cincinnati College of Medicine, OH, USA; 5 University of Notre Dame, IN, USA

**Keywords:** *P* factor, the general factor of psychopathology, bifactor, bifactor-(*S*−1), anomalous results, collapsing factors

## Abstract

Symmetrical bifactor models are frequently applied to diverse symptoms of psychopathology to identify a general *P* factor. This factor is assumed to mark shared liability across all psychopathology dimensions and mental disorders. Despite their popularity, however, symmetrical bifactor models of *P* often yield anomalous results, including but not limited to nonsignificant or negative specific factor variances and nonsignificant or negative factor loadings. To date, these anomalies have often been treated as nuisances to be explained away. In this article, we demonstrate why these anomalies alter the substantive meaning of *P* such that it (a) does not reflect general liability to psychopathology and (b) differs in meaning across studies. We then describe an alternative modeling framework, the bifactor-(*S*−1) approach. This method avoids anomalous results, provides a framework for explaining unexpected findings in published symmetrical bifactor studies, and yields a well-defined general factor that can be compared across studies when researchers hypothesize what construct they consider “transdiagnostically meaningful” and measure it directly. We present an empirical example to illustrate these points and provide concrete recommendations to help researchers decide for or against specific variants of bifactor structure.

Many authors use bifactor models in attempts to identify overarching vulnerability multiple domains of psychopathology (e.g., internalizing, externalizing, thought disorders [TDs]) have in common (e.g., Carragher et al., 2016; Caspi et al., 2014; Gomez et al., 2019; Haltigan et al., 2018; Laceulle et al., 2015; Lahey et al., 2012, 2018; Markon, 2019; Martel et al., 2017; Smith et al., 2020; Swales et al., 2020; Tackett et al., 2013). Bifactor models structure psychopathology in terms of (a) a general factor on which all indicators of all domains load and (b) domain-specific factors that depict variance not shared with the general factor or by one another. In clinical psychology and psychiatry, the general factor has been referred to as the “general factor of psychopathology” or “*P* factor” (e.g., Caspi et al., 2014). *P* is often assumed to be transdiagnostic in the most general sense—in other words, a latent manifestation of a single causal factor associated with all symptoms of most mental disorders.

The growing number of bifactor applications in psychopathology research suggests that modeling a general factor provides a major advantage over models with correlated factors. The term “general factor of psychopathology” suggests a clear and substantively meaningful interpretation. Initially identified by Lahey et al. (2012), *P* is typically interpreted as an overarching vulnerability to psychopathology derived from a common etiological mechanism. Many authors, including Caspi et al. (2014), attribute *P* to deficiencies in frontally mediated executive processes, such as inhibitory control and self-/emotion regulation (see, for example, Beauchaine & Cicchetti, 2019; Martel et al., 2017). According to such theories, compromised executive and self-regulatory abilities potentiate virtually all forms of psychopathology including internalizing, externalizing, and thought problems through failures in top-down cortical inhibition of subcortical neural systems (see Beauchaine & Zisner, 2017, and Carver et al., 2017, for related interpretations and see Smith et al., 2020 for alternative conceptualizations).

Although the *P* factor has generated lively discussions over shared etiological mechanisms of psychopathology, these discussions assume that the general factor is a valid representation of shared liability. Recently, evidence has emerged that questions this assumption. It is, therefore, unclear to what extent the general factor in bifactor models indeed represents general psychopathology—or whether it measures something different. This ambiguity is one reason why the increasing use of bifactor models to depict psychopathology has been critically scrutinized by several authors (e.g., Aristodemou & Fried, 2020; Bonifay et al., 2017; Burns et al., 2020a; Eid et al., 2017; Heinrich et al., 2020; Levin-Aspenson et al., 2021; Sellbom & Tellegen, 2019; van Bork et al., 2017; Watts et al., 2019). According to these authors, it is crucial to define conditions under which the general factor can be interpreted in an unambiguous and meaningful manner.

In this manuscript, we argue that commonly applied symmetrical bifactor models (BF_SYM_, Holzinger & Swineford, 1937) are of limited use for modeling *P* and that the more theory-oriented bifactor-(*S*−1) approach (BF_*S*−1_, Eid et al., 2017) is often preferred. Given anomalous results, which often emerge from symmetrical bifactors models (see below), *P* typically does not depict an overarching factor of psychopathology, as researchers intend, but instead carries a meaning that varies from study to study (e.g., Levin-Aspenson et al., 2021; Watts et al., 2020). The BF_*S*−1_ approach follows a logic that differs from that currently used in *P* factor research to address these issues. Instead of modeling a broad general psychopathology factor and trying to ascertain what this factor depicts, those who apply the BF_*S*−1_ approach (a) define the transdiagnostic construct *a priori* and (b) measure it directly. In doing so, BF_*S*−1_ models avoid ambiguities of BF_SYM_ models, providing a general factor that is well defined, replicable, and comparable across studies.

Thus, we agree that general vulnerabilities are useful explanatory constructs for characterizing the emergence and persistence of various mental disorders. However, we argue that BF_SYM_ models typically do not meaningfully depict these communalities but rather leave researchers with results difficult to compare, accumulate, and generalize. We illustrate our arguments by means of various published bifactor models in *P* factor research. Our aim is not to criticize these studies or authors (indeed, some of us are guilty of the same misinterpretations) but to contribute to a better understanding of the meaning(s) and interpretation(s) of the general factor as depicted in bifactor models.

## Symmetrical Bifactor Models

**Figure 1:**
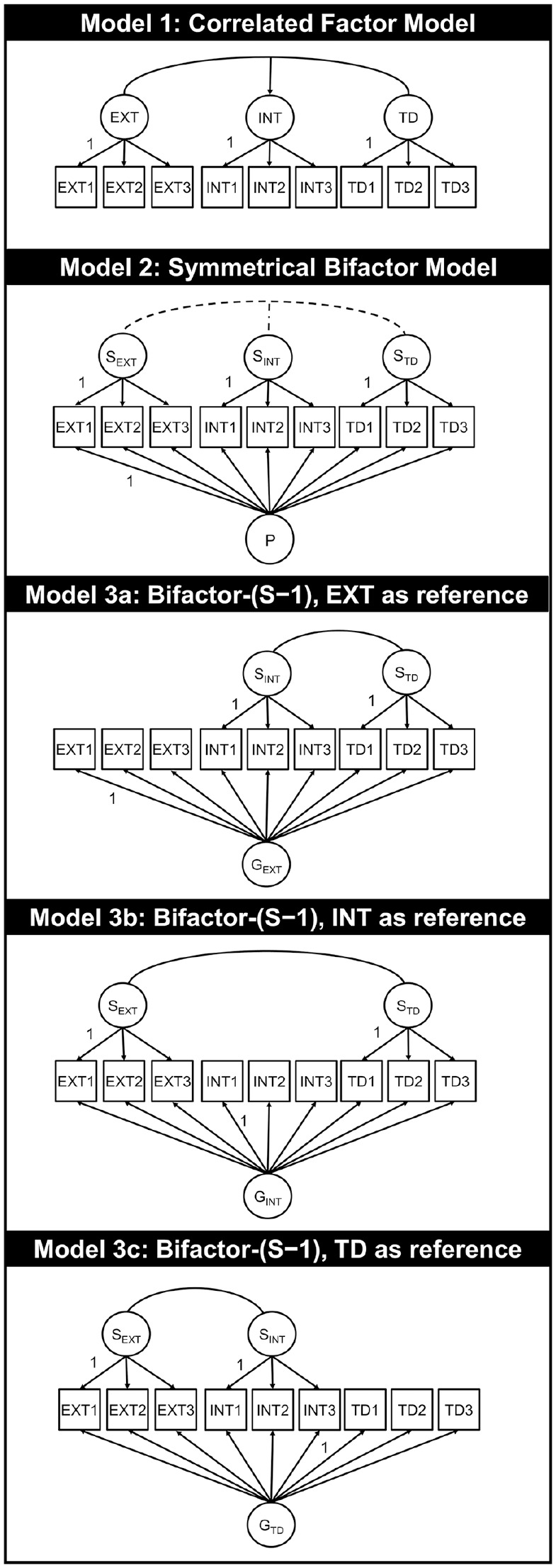
Different Models for Three Items Assessing Internalizing (INT1–INT3), Externalizing (EXT1–EXT3), and Thought Disorders (TD1–TD3) *Note.* Model 1: Correlated factor model: Each item loads on one domain-specific first-order factor and all factors are correlated. Model 2: A fully symmetrical bifactor model: Each item loads on one specific factor as well as on the general factor. The dotted line indicates correlations, which are allowed in many empirical applications but are inadmissible and should be avoided. Model 3a to Model 3c: Bifactor-(*S*−1) model: Each item loads on the general factor. Items, which do not belong to the reference domain, also load on one specific factor. INT = internalizing; EXT = externalizing; TD = though disorders.

When clinical researchers think about bifactor structures, they usually have BF_SYM_ models in mind (see Figure 1, Model 2 for an example). In these models, each item or scale loads on a general factor—the general factor of psychopathology—and a domain-specific *S* factor (Eid et al., 2017; Holzinger & Swineford, 1937; Markon, 2019). Thus, the variance of each observed indicator is decomposed into three parts. The first is determined by the general factor and must be different from zero. Otherwise, the item has nothing in common with the general factor of psychopathology that is supposed to underlie all symptoms. The second part is variance due to a narrower domain-specific factor. Since each item should also represent a specific domain of psychopathology (e.g., internalizing or externalizing), this part must be meaningfully different from zero as well. The third part comprises measurement error.

*S* factors are residual factors with a mean of zero that capture deviations of domain-specific values from values expected based on the general factor (e.g., Eid et al., 2017; Reise, 2012). Correlations between *S* factors must be fixed to zero in the BF_SYM_ approach. This zero-correlation follows from the assumption that the general psychopathology factor causes different domains of psychopathology to correlate. Those correlations should, therefore, vanish when effects of the general factor are removed (Eid et al., 2017; Reise, 2012). Reise (2012) point out that correlations between *S* factors contradict the idea of a single unifying factor because they suggest “the presence of additional and unmodeled general factors” (p. 692). Furthermore, the general factor and all *S* factors are uncorrelated by definition (Eid et al., 2017; Holzinger & Swineford, 1937; Markon, 2019; Reise, 2012).

Many clinical researchers consider BF_SYM_ models as theoretically and/or empirically superior to correlated first-order factor models (see Figure 1, Model 1) because they appear to include a single overarching dimension that explains why dimensions of psychopathology and mental disorders co-occur (i.e., comorbidity). In addition, BF_SYM_ models often provide a better fit than competing models without a higher order *P* factor. However, several authors have cautioned against relying on fit indices when deciding whether or not to use BF_SYM_ models, given that such models often provide a better fit whether or not they are correct (e.g., Bonifay & Cai, 2017; Greene et al., 2019; Murray & Johnson, 2013; Sellbom & Tellegen, 2019).

Furthermore, in clinical research, several BF_SYM_ models yield solutions that contain improper (“inadmissible”) parameter estimates (e.g., negative residual variances or negative specific factor variances; Caspi et al., 2014; Romer et al., 2018, 2021) and/or otherwise anomalous results that are either unexpected (e.g., specific factor loadings that are very small or fixed to zero; Brandes et al., 2019; Lahey et al., 2012; Snyder et al., 2017; Tackett et al., 2013), difficult to interpret, or fully uninterpretable (e.g., negative factor loadings, Castellanos-Ryan et al., 2016; Gluschkoff et al., 2019; Martel et al., 2017; Watts et al., 2019).

As we detail below, such results challenge the assumption that *P* represents a general factor that underlies all symptoms, often redefining *P* empirically as a specific domain of psychopathology (i.e., internalizing, externalizing, *or* TDs), depending on sample-specific solutions. However, even without anomalous results, the general factor in BF_SYM_ models often lacks a clear interpretation because domains of psychopathology are not interchangeable, a point we return to and elaborate below.

## Anomalous Results in *P* Factor Studies

When modeling a general psychopathology factor using BF_SYM_ models, *S* factors sometimes “collapse.” This occurs when an *S* factor has a very small or negative variance estimate and/or when many of the standardized loadings on the *S* factor are close to zero (e.g., ≤ .2) and/or nonsignificant. In other cases, *S* factors “partially collapse.” This occurs when only a few indicators have substantial loadings on that *S* factor. In cases of fully and partially collapsing *S* factors, the factor in question may not exist. In this case, indicators that should depict both (a) transdiagnostic vulnerability as expressed by the general *P* factor and (b) a specific domain of psychopathology, instead measure *only P*. Moreover, irrespective of whether an *S* factor collapses entirely or partially, the interpretation of the general factor is the same—it represents the construct underlying the indicators that load exclusively on the general factor. We explain this in detail shortly.

For example, Caspi et al. (2014) constructed a BF_SYM_ model with three correlated *S* factors (internalizing, externalizing, TD) and reported estimation problems. Caspi et al. (2014) dropped the TD factor from their model to deal with this inadmissible estimate. Romer et al. (2021, see also Romer et al., 2018) did the same, reporting a Heywood case for the mania indicator after including the general factor in a model that used the same set of *S* factors as Caspi et al. (2014). Like Caspi et al. (2014), Romer et al. (2018) omitted the specific TD factor. Thus, instead of *P* being a general factor underlying all 11 symptom dimensions in the study, the general factor became a TD factor (represented by obsessive-compulsive disorder (OCD), mania, and schizophrenia) in both studies. We detail why this occurs and elaborate on the resulting problems with interpretation shortly.

Lahey et al. (2012, their Figure 1) also examined three domains of psychopathology (distress, fear, externalizing) with a BF_SYM_ model. The generalized anxiety disorder (GAD) and agoraphobia/panic (AP) indicators did not load substantially on their respective *S* factors (standardized loadings: GAD = .13; AP = .16), but both indicators loaded strongly on the general factor (GAD = .85 and AP = .77). Thus, although the corresponding *S* factors did not collapse entirely, certain indicators measured only the general factor and no longer measured a specific *S* factor. As we explain below, these findings indicate that *P* was in fact a GAD/AP factor—not a general liability factor (see Castellanos-Ryan et al., 2016 for a similar example with a partially collapsing externalizing factor).

In addition, it is challenging to properly interpret one or more S factors in some bifactor applications because patterns of factor loadings differ from those expected based on both theory and the correlated factors model. For example, if all factor loadings in a correlated factors model have a positive sign (e.g., when all are symptoms of attention-deficit/hyperactivity disorder [ADHD]), they should all have the same sign even after the general factor is included. Instead, factor loadings in some applications inconsistently change signs (some from positive to negative while others remain positive). Changing signs of factor loadings demonstrate that indicators of the same facet behave differently with respect to their *S* factor after the general factor is added. The meaning of the *S* factor changes, which should not happen if indicators of the facet are homogeneous and *interchangeable* (see below). Although there are no statistical reasons why all factor loadings of a facet should change signs consistently, such unexpected patterns typically lack theoretically sound explanations (Eid et al., 2017). For example, in applying a BF_SYM_ model with uncorrelated *S* factors to fear, distress, and externalizing, Watts et al. (2019) reported a negative loading (–.34) for the general anxiety indicator on its specific distress factor. In contrast, the same indicator had a strong positive loading on the general factor (.84). The only other indicator of the distress-specific factor (major depression) loaded moderately (.36) on the same *S* factor but loaded .77 on the *P* factor. In addition, generalized anxiety and major depression were the only indicators that loaded highly on the general factor (all other loadings were ≤ .49). These findings render the specific distress factor difficult to interpret, and any interpretation of *P* as a general factor is questionable (here, the general factor was defined primarily by generalized anxiety and major depression indicators, which did not have a stable *S* factor).

Anomalous results are not specific to BF_SYM_ models intended to characterize *P*. In their review of 82 bifactor studies across different areas of psychology, Eid et al. (2017) found anomalous results in at least 50 (61%) of applications. Similar problems frequently occur in applications of the bifactor approach to research on depression (Heinrich et al., 2020). Burns et al. (2020a) examined 24 bifactor applications of ADHD symptoms or ADHD/oppositional defiant disorder (ODD). Among these 24 studies, over 75% yielded anomalous loading patterns or inadmissible solutions. Researchers who use BF_SYM_ models in psychopathology research should be aware of anomalous results and resulting interpretative challenges. In the next section, we provide a more detailed explanation of why anomalous results change the meaning of a general factor.

### Why Collapsing Factors Change the Meaning of a General Factor

As described above, a problem in applications of BF_SYM_ models of general psychopathology is that one *S* factor has weak, inconsistent, and/or negative loadings, or a variance estimate that is zero or close to zero. Fully collapsing specific factors in BF_SYM_ models change indicators of the collapsing factor into “pure” indicators of *P—*not indicators of a specific factor. Consequently, the meaning of the general factor is defined completely by specific *S* factor items, and the general factor becomes equivalent to the corresponding first-order *S* factor for the collapsing facet (see Supplemental Material 1 for an extended discussion). Thus, the “general” *P* factor is no longer a general factor of psychopathology. Instead, it is an empirically defined latent variable underlying indicators of the collapsed factor. This is similar to an exploratory factor analysis in which items that have no cross-loadings but load strongly on one factor are considered putative markers of that construct. In other words, *one could depict the latent variable one consider to be the general factor by using only indicators that load exclusively on the general factor and omitting the rest of the model*.

The fact that the “general” *P* factor in models with collapsing specific factors is no longer interpretable as a general factor can be readily seen through the measurement equation of a BF_SYM_ model with orthogonal *S* factors. Let us assume that the model contains a specific TD factor in addition to a specific internalizing and externalizing factor, and that we use sum scores that measure the severity of mania (MAN), OCD, and psychosis as indicators of the TD factor. The bifactor measurement equation for mania is given by $MAN=\alpha_{\mathrm{MAN}}+\lambda_{P,\;\mathrm{MAN}}\times P+\lambda_{S\_\mathrm{TD},\;\mathrm{MAN}}\times S_{\mathrm{TD}}+\varepsilon_{\mathrm{MAN}}$ . That is, the observed score of mania is the sum of an intercept $\left(\alpha_{\mathrm{MAN}}\right)$ , the general factor score weighted with the factor loading of mania on *P* $\left(\lambda_{P,\;\;\mathrm{MAN}}\right)$ , the *S* factor score weighted by the factor loading of mania on the specific TD factor $\left(\lambda_{S\_\mathrm{TD},\;\;\mathrm{MAN}}\right)$ , and measurement error $\left(\varepsilon_{\mathrm{MAN}}\right)$ .

When $S_{\mathrm{TD}}=0$ (i.e., the variance of the *S* factor is zero or nonsignificant/very small), $\lambda_{S\_\mathrm{TD},\;\;\mathrm{MAN}}$ = 0 (i.e., the factor loading of mania on the specific TD factor is fixed to zero or nonsignificant/very small), or when $\lambda_{S\_\mathrm{TD},\;\;\mathrm{MAN}}\times S_{S\_\mathrm{TD}}$ is dropped because researchers omit the specific TD factor, the measurement equation reduces to $MAN=\alpha_{\mathrm{MAN}}+\lambda_{P,\;\;\mathrm{MAN}}\times P+\varepsilon_{\mathrm{MAN}}$ . The observed mania score is now the sum of an intercept $\left(\alpha_{\mathrm{MAN}}\right)$ , the “general” factor weighted with the factor loading of mania on *P* $\left(\lambda_{P,\;\;\mathrm{MAN}}\right)$ and measurement error $\varepsilon_{\mathrm{MAN}}$ . The general factor is now the true score variable pertaining to mania. It differs from mania only in terms of an intercept $\left(\alpha_{\mathrm{MAN}}\right)$ , a scaling constant (factor loading $\lambda_{P,\;\;\mathrm{MAN}}$ ) and random measurement error $\left(\varepsilon_{\mathrm{MAN}}\right)$ .

These equations show that when an *S* factor fully collapses, that is, if all indicators have non-significant loadings on the *S* factor, or the *S* factor shows a zero or negative variance estimate, the general factor becomes a common factor pertaining to mania and/or the other indicators of that *S* factor. In our example, the general factor becomes a TD factor—a latent variable underlying severity scores of mania, psychosis, and OCD. Notice, however, that the general factor can also have a more narrowly defined meaning. If only the *S* factor loading of the mania indicator becomes non-significant, with factor loadings of OCD and psychosis on *P* and *S* remaining strong, the general factor depicts just mania. This makes clear why the meaning of *P* changes depending on which *S* factor collapses, is weakly defined, or omitted entirely. This underscores why the general factor is not *P* even if all indicators load on it. Instead, the meaning of the general factor is defined by indicators that load *only* on it and not on other factors. Consider a study in which a growth curve model is used to model change in depression severity across several measurement occasions. All depression severity scores load onto the intercept factor with the same loading of 1.0. The intercept factor is interpreted correctly as the severity of depression pertaining to the time point for which the slope factor loading is fixed to zero and *not* as a general factor of depression severity. Similarly, *P* is only interpretable as a general factor when all variables have substantial *P and* substantial *S* factor loadings. We illustrate this issue in more detail in the empirical section below.

Our example used to illustrate the measurement equation can be transferred to results reported by Caspi et al. (2014). In that application, the specific TD factor was dropped completely from the model due to a negative variance estimate. Therefore, the *P* factor was no longer a general factor but instead became a TD factor. This is demonstrated by the fact that mania and schizophrenia indicators (a) loaded particularly highly on *P* (.97 and .82; the *P* factor loading for mania was highest of all indicators) and (b) had no additional specific factor. Caspi et al. (2014) provided further evidence that both latent variables were the same. Standardized factor loadings of indicators of the TD factor were very similar in the model with correlated factors and in the bifactor model (.73, .98, and .83 vs .73, .97, and .82). Moreover, factor scores of the *P* factor and the TD factor showed a correlation of .997, and correlations between the *P* factor and external variables were similar in magnitude to correlations between the first-order TD factor and external variables in the model with correlated factors. In fact, the average deviation of absolute values of correlations across all tested associations was only .007, and there was no absolute difference larger than .017.

Caspi et al. (2014) argued that symptoms of TDs are indicators of *P*. However, this interpretation leads to a conceptual problem because the *P* factor and the TD factor are the *same* variables. As explained above, Caspi et al. could have modeled what they considered to be the *P* factor if they had used only the indicator for mania, obsessive-compulsive disorder, and psychosis, omitting the rest of the model (we illustrate this in detail in our empirical example below and provide an additional example in the supplemental material). Thus, if we label the general factor as *P* in the bifactor model, we should also label the TD factor in the correlated factors model as *P* (because they are the same variables). Alternatively, we could label *both* latent variables as TD factors. Giving the same latent factor different labels suggests substantive differences where none exists (reconsider the correlation of .997 between the *P* and *S* factor scores).

The same arguments apply to studies in which *S* factors are omitted post hoc. For example, Romer et al. (2021) favored a BF_SYM_ model with no *S* factor for TD and, like Caspi et al. (2014), found an extremely high correlation between *P* and the TD *S* factor they identified in their correlated factors model (.98). This shows that Romer et al. (2021) tested the relationship between neocortical thickness and TD and *not* necessarily the relationship between neocortical thickness and an overarching *P*.

Similarly, in one application reported by Tackett et al. (2013), *S* factor loadings for major depressive disorder (MDD) and GAD were omitted from their BF_SYM_ model (see their Figure 3), making the general factor an MDD/GAD factor (MDD and GAD loaded .94 and .95 on the general factor, respectively). That factor might carry a similar (but not interchangeable) meaning as the general factor modeled in Brandes et al. (2019). In that study, items for withdrawn-depressed and MDD loaded exclusively on *P*, making the general factor a depressive mood factor.

In all previously cited studies, entire *S* factors were omitted or *S* factor loadings that were nonsignificant were fixed to zero. However, the same shift in interpretation occurs when one or more indicators have no substantial loadings on their *S* factors but load highly on the general factor, as in both the Lahey et al. (2012) and Watts et al. (2019) applications. In both cases, *S* factors did not collapse completely, but several indicators had strong loadings only on the general factor and not on their *S* factors. Similarly, Martel et al. (2017) modeled general factors of psychopathology among children and their mothers. In each model, at least one item had a loading of zero or close to zero on one of the *S* factors. When modeling *P* in children, the *S* factor loading of the autism spectrum indicator was fixed to zero, making the general factor in children an autism factor. Modeling maternal *P*, the loading of the separation anxiety indicator on the *S* factor for fear was very close to zero (−.006), giving the maternal general factor the meaning of separation anxiety. Consequently, there was no consistency in the meaning of *P* across groups (or studies).

### Why Collapsing Factors Also Change the Meaning of *S* factors

As alluded to above, when an *S* factor collapses, the meaning of the remaining *S* factors also changes. Given that the general factor is now defined based on indicator(s) with no *S* factor (or zero *S* factor loadings), the remaining *S* factors are comprised of specific variance relative to a general factor. For example, the *S* factor for internalizing in Caspi et al. (2014) now indicates variance in the specific internalizing factor that is *independent* of the TD factor rather than specific internalizing variance independent of a general *P* factor. The same is true for the Romer et al. (2021) application. In Brandes et al. (2019), the specific internalizing factor represents the part of internalizing that is not predicted by depressive mood, and in Tackett et al. (2013), the internalizing factor represents specific internalizing variance that is independent of the latent variable underlying MDD/GAD. Thus, the meaning of *S* factors also varies from study to study when using the BF_SYM_ approach.

In our experience, even though weak or collapsed *S* factors (or individual variables without substantial *S* factor loadings) occur in empirical applications, researchers continue to interpret *P* and *S* in the same way as if all variables had substantial loadings on both *P* and *S* factors. That is, researchers continue interpreting *P* as a general factor and *S* factors as residuals with respect to the general factor. As we have shown above, such an interpretation is unwarranted and (however unintentionally) misleading when *S* factors collapse or when *S* factor loadings are zero or near zero. Next, we describe the distinction between interchangeable and structurally different domains and explain why this differentiation is crucial for selecting an appropriate psychopathology bifactor model.

## Interchangeability as a Prerequisite for Meaningful General (*P*) Factors

Eid and Koch (2014) argue that, from the perspective of stochastic measurement theory, *interchangeability* of domains is an essential prerequisite for a properly interpretable general factor in BF_SYM_ models and for avoiding anomalous results, which, as described above, are common in empirical applications (see also Eid et al., 2017). Interchangeability requires a universe of domains from which a small subset of those domains is selected randomly. Domains represent *random effects* when interchanging any domain for another does not alter the meaning of the general factor, and when each combination of domains is equally well suited to represent the general factor.

Interchangeability can be attained, for example, when researchers randomly sample situations from a person’s life and ask the person to evaluate his or her depressive mood on several items in each specific situation. When such ratings are modeled as a bifactor structure, the general factor represents expected depressive mood across situations, and *S* factors represent situation-specific deviations. The same applies when researchers randomly select friends from an individual’s social network and ask each friend to assess how depressed the person is. The general factor represents that person’s average depressive mood as perceived by their friends, and *S* factors capture informant-specific deviations. Both examples represent a two-level measurement design with measurements nested within persons (Eid et al., 2017; Eid & Koch, 2014; Geiser et al., 2012). A random sampling at both levels (persons and situations) ensures that the general factor is appropriately interpretable as an expected value across domains (Eid et al., 2017; Eid & Koch, 2014). This sampling process implies that it does not matter which random sample of situations or a random sample of friends we choose—an idea that is may be impossible when measuring symptoms or domains of psychopathology. Instead, it seems more reasonable to assume that symptoms and domains of psychopathology are *structurally different* and that each combination of symptoms provides a unique perspective on the individual’s burden. Being structurally different also means that domains can have domain-specific vulnerabilities and can impair psycho-social functioning differently. Thus, even when no anomalous results occur, we consider BF_SYM_ models are limited for modeling general psychopathology. Although it is always possible to model a general factor underlying structurally different correlated facets of psychopathology, the resulting general and *S* factors are challenging to interpret whenever facets lack interchangeability and do not share the same nomological net (Eid et al., 2017; Lee & Cadogan, 2013).

The fact that different domains of psychopathology are structurally different is reflected empirically in inter-domain correlations that are often heterogeneous in magnitude. In Caspi et al. (2014), for example, latent correlations differed strongly between the three first-order factors (.33 between internalizing and externalizing factors, .85 between internalizing and TD, .58 between externalizing and TD; see also Laceulle et al., 2015; Romer et al., 2018, 2021). For interchangeable domains, we expect these intercorrelations to be about equal. This is because sampling and measurement error are the only sources of dispersion around the sampling mean for interchangeable domains; there is no systematic structural difference between interchangeable domains that would cause some correlations to be substantially higher than others. In contrast, structurally different domains (e.g., internalizing, externalizing, TD) differ systematically from one another, leading to heterogenous inter-domain correlations. They provide different information about different facets of psychopathology.

Geiser et al. (2015) showed that inadmissible results in B_SYM_ models are more likely when models are fitted to structurally different domains. Therefore, the frequently encountered anomalous results in *P* factor studies are probably best understood as a result of applying a modeling approach that requires interchangeable domains to structurally different domains. Preferably, we could select a modeling approach that (a) considers structural differences, (b) avoids anomalous results, and (c) gives the general factor unambiguous meaning. We now present the BF_*S*−1_ approach as such an alternative.

## Bifactor-(*S*−1) Models and the Meaning of *P* and *S*

The BF_*S*−1_ approach was introduced as an alternative for estimating bifactor models and is designed specifically to account for structurally different domains (Eid et al., 2017). The major difference between the BF_SYM_ model and the BF_*S*−1_ model is that the latter contains a subset of items (reference domain) which load exclusively on the general factor as a starting point (see Figure 1). The remaining items (pertaining to nonreference domains) load on the general factor *and* one factor. This structure ensures that both general and *S* factors are unambiguous in their psychometric definition and interpretation.

In BF_*S*−1_ models, the factor labeled “general” does not represent an overarching dimension—even though all items load on it. The meaning of the general factor is instead defined *a priori* by items that pertain to the reference domain, that is, items that load exclusively on the general factor. To avoid confusion with *P*, which is inextricably linked to “general psychopathology,” we refer to a general factor in a BF_*S*−1_ model hereafter as *G_i_*, where *i* denotes the latent variable underlying the indicators of the reference facet. Take Model 3c, depicted in Figure 1, as an example. Items assessing TD represent the reference domain. Thus, the general factor measures TD (*G*_TD_) and, most importantly, does so in the same manner as in the model with correlated first-order factors. The general factor and the corresponding first-order factor are equivalent—they depict the same latent variable (see Supplemental Material 1, see also Eid et al., 2017 for a more formal presentation; see also Burns et al., 2020b; Geiser et al., 2008, 2015; Heinrich et al., 2020). The BF_*S*−1_ approach, therefore, makes specific use of the fact that items loading only on the general factor define its meaning. In the BF_*S*−1_ model, the general factor is defined *a priori* by selecting a theoretically meaningful reference domain.

The meaningfulness of the reference domain depends on indicators used to depict that reference. In principle, it is possible to use heterogeneous indicators. However, latent variables that underlie structurally different indicators may exhibit the same problems as general factors that underlie structurally different facets: the derived latent variables can be challenging to interpret and compare unless different studies use identical or interchangeable indicators in combination with the same measurement model. Therefore, BF_*S*−1_ models are most informative if indicators approach the ideal of reflective indicators. Such indicators are conceptually interchangeable, unidimensional, show high standardized factor loadings on their facet factor, and share the same nomological net (e.g., Bollen & Bauldry, 2011; Bollen & Lennox, 1991; Jarvis et al., 2003).

Selecting a reference facet and thinking carefully about appropriate reference indicators is different from the BF_SYM_ approach in which data decide what the general factor represents. Consequently, as we explained above, the meaning of *P* in a BF_SYM_ model depends largely on which set of structurally different domains is included in a given study and on which domains collapse.

When selecting a reference domain a priori, the psychometric definition and meaning of the general factor are clear: *G*_i_ represents common true score variance reflected in indicators of the reference domain (e.g., TD symptoms). The meaning and interpretation of *G*_i_ do not change when other domains are added to the model or when domains are removed from the model. As long as the reference domain remains the same, *G*_i_ has the same meaning across studies that include different domains as *S* factors.

However, it is essential to keep the following characteristic of the BF_*S*−1_ approach in mind: *Whenever researchers change the reference facet, they also change the meaning of the general factor and specific factors.* For example, reconsider the study of Caspi et al. (2014). The authors removed the TD factor. Consequently, the general factor represents the latent variable underlying mania, OCD, and psychosis (*G*_TD_). Alternatively, they could have removed the specific internalizing factor. In that case, the general factor would have represented the latent variable underlying the internalizing indicators (*G*_INT_). Similarly, if they had removed the specific externalizing factor, the general factor would have represented externalizing (*G*_EXT_). *G* would have had a completely different meaning in each model.

This characteristic of the BF_*S*−1_ approach has significant implications. Researchers who define a reference facet a priori cannot replace that facet without changing the meaning of the model. Moreover, researchers cannot compare their results with other studies unless the same or an empirically interchangeable reference facet is used. This feature of BF_*S*−1_ models underlines why only theory and not model fit is a valid means of selecting a reference facet (Burns et al., 2020a; Eid et al., 2017; Geiser et al., 2008, 2012; Heinrich et al., 2020). Using model fit as a guide can and does lead to models with different reference facets and a nonaccumulative scientific practice (as is the case with current *P* factor research). Consequently, the BF_*S*−1_ approach is most helpful when researchers (a) hypothesize a priori what construct they think is “transdiagnostically meaningful,” (b) measure the construct directly, (c) hypothesize how their understanding of “transdiagnostically meaningful” translates into estimated parameters, and (d) test these expectations with empirical data.

*S* factors in BF_*S*−1_ models are clearly defined as residual factors with a mean of zero (Eid et al., 2017). These *S* factors offer a cleaner interpretation than those of BF_SYM_ models. They represent the part of a domain that cannot be explained by the reference facet—not the part of a domain that cannot be explained by something that researchers do not know what it actually is, as is the case with *P*. Take Model 3c, depicted in Figure 1, again as an example. The TD factor represents the reference domain; therefore, the *S* factor for internalizing represents that part of internalizing that is not be predicted linearly by TD (*G*_TD_). However, when internalizing is used as reference (*G*_INT_, see Figure 1, Model 3b), the general factor depicts internalizing, and the *S* factor for TD represents that part of TD that cannot be predicted linearly by internalizing. Thus, changing the reference facet changes the meaning of the general factor *and* the meaning of the *S* factors.

We explained earlier that correlations between *S* factors in BF_SYM_ models must be fixed to zero. In contrast, these correlations can be estimated and meaningfully interpreted in BF_*S*−1_ models. These associations are partial correlations, representing strengths of associations between first-order factors, corrected for the influence of the reference domain (Eid et al., 2017; Geiser et al., 2008). They, therefore, represent what two *S* factors have in common once the effect of the reference domain is partially out. For example, the correlation between the *S* factors for internalizing and externalizing reported by Caspi et al. (2014) shows that internalizing and externalizing share variance above-and-beyond what both domains share with TD.

## Illustrative Example

We now illustrate the effect of collapsing factors on the meaning of the general factor in the BF_SYM_ model, as well as fundamental properties of BF_*S*−1_ models based on an empirical example (for other applied examples, see Burns et al., 2020a; Demkowicz et al., 2020; Gäde et al., 2017; Greene et al., 2021; Haywood et al., 2021; Heinrich et al., 2020; Hoffmann et al., 2021; Junghänel et al., 2020). First, we show that the BF_SYM_ model produces anomalous results and that the general factor in the BF_SYM_ model becomes the specific latent variable underlying indicators of a collapsing factor. Second, we show it does not matter which *S* factor is omitted to avoid anomalous results, but that selection of the omitted *S* factor determines the meaning of the general factor. Last, we illustrate that, provided the reference domain of a BF_*S*−1_ model remains the same, the meaning of the general factor also remains the same, regardless of whether domains are added or removed.

We use data originating from an ongoing study in which we aim to construct a scale to allow *individual symptoms* of depressive disorders to be represented as latent variables. Each symptom is assessed with several items. Participants rated how often they experienced emotions and behaviors described in the items in the 2 weeks preceding assessment using a rating scale ranging from *never* (0) to *always* (5). Categories 4 and 5 were collapsed because the category *always* was rarely endorsed for the indicators assessing low appetite. Items were taken from the *Inventory of Depression and Anxiety Symptoms* (Watson & O’Hara, 2017; Watson et al., 2008, 2012) and were complemented by additional items unless several items were available to assess a specific symptom.

Participants were also encouraged to assess their sleep quality (“How restful was your sleep?”), rated on a 10-point scale with higher values indicating better sleep quality. In addition, we used an item from the stress module of the Patient Health Questionnaire (Löwe et al., 2004) asking participants how much they felt impaired due to stress at work/school in the 4 weeks preceding assessment. That item was rated on a 3-point scale ranging from *not at all* (0) to *severely impaired* (2). All self-report questionnaires were completed online. The sample consisted of 450 persons with an average age of 25.6 years (*SD* = 7.5; range: 18–62). Most participants were female (*n* = 363, 81%). Data collection was approved by the Ethics Committee of the Freie Universität Berlin.

All models presented below were estimated using the weighted least squares means and variance adjuste (WLSMV) estimation method implemented in M*plus* version 8.3. Indicators of symptoms and the item assessing stress at work were treated as ordered categorical. Sleep quality was treated as a continuous variable. Output files, including descriptive item statistics, are available online (https://osf.io/sq4zd/). An additional empirical example illustrating how collapsing factors affect the interpretation of the latent variables in BF_SYM_ models is provided in Supplemental Material 2.

### Symmetrical Bifactor Models

For the first part of the illustrative example, we use symptoms of sadness, low appetite, and concentration problems (each assessed with three items) and estimate a correlated factor model. We also test a BF_SYM_ model with uncorrelated *S* factors.

#### Model Fit

Both models fitted the data well (see Table 1). As is typical in empirical applications (see above), fit of the BF_SYM_ model was superior to fit for the model with correlated factors. This is to be expected because the BF_SYM_ model is less restrictive (estimates more free parameters).

**Table 1.** Model Fit.

Model	χ²	*df*	*p*	RMSEA	[90% CI]	CFI	SRMR
CFM _SAD, LAP, CON_	45.28	24	.005	.044	[.024, .064]	.998	.023
BF_SYM, SAD, LAP, CON_	23.42	18	.175	.026	[.000, .052]	.999	.015
BF_*S*−1_, _SAD, *S*-LAP, *S*-CON_	27.17	20	.131	.028	[.000, .053]	.999	.017
BF_*S*−1_, _LAP, *S*-SAD, *S*-CON_	31.30	20	.051	.035	[.000, .058]	.999	.018
BF_*S*−1_, _CON, *S*-SAD, *S*-LAP_	40.23	20	.005	.047	[.026, .069]	.998	.020
BF_*S*−1_, _SAD, *S*-EAR, *S*-DES_	24.86	20	.207	.023	[.000, .049]	1.000	.014
CFM _SAD, LAP, CON_ + stress + sleep quality	59.76	36	.008	.038	[.020, .055]	.998	.021
BF_SYM, SAD, LAP, CON_ + stress + sleep quality	dnc.						
BF_*S*−1_, _SAD, *S*-LAP, *S*-CON_ + stress + sleep quality	41.10	32	.130	.025	[.000, .045]	.999	.017
BF_*S*−1_, _LAP, *S*-SAD, *S*-CON_ + stress + sleep quality	45.35	32	.059	.030	[.000, .049]	.999	.017
BF_*S*−1_, _CON, *S*-SAD, *S*-LAP_ + stress + sleep quality	54.99	32	.007	.040	[.021, .057]	.998	.019
BF_*S*−1_, _SAD, *S*-EAR, *S*-DES_ + stress + sleep quality	47.81	32	.036	.033	[.009, .052]	.999	.017

*Note.* RMSEA = root mean square error of approximation; CI = confidence interval; CFI = comparative fit index; SRMR = standardized root mean residual; CFM = correlated factor model; BF = bifactor; BF_*S*−1_ = bifactor-(*S*−1) model; *df* = degrees of freedom; dnc. = did not converge; Facets: SAD = sadness; LAP = low appetite; CON = concentration problems; SYM = symmetrical bifactor model; EAR = early awakening; DES = problems in decision-making.

#### Correlations

Correlations between factors in the correlated factor model are summarized in Table 2. The correlation between the sadness and concentration factors was rather high (.74), whereas correlations between the sadness and the low appetite factors (.52) and between the low appetite and concentration factors (.40) were substantially lower. *This pattern indicates that the three domains are not interchangeable but are structurally different*. For interchangeable domains, one would expect correlations to be very similar.

**Table 2.** Factor Loadings of the Correlated Factor Model, the Fully Symmetrical Bifactor Model, as well as the Bifactor-(S−1) Model With Different Reference Domains.

Factor	SFM	CFM	BF_SYM_		BF_*S*−1_		BF_*S*−1_		BF_*S*−1_		Factor	BF_*S*−1_	
					SAD_*S*-LAP, *S*-CON_		LAP_*S*-SAD, *S*-CON_		CON_*S*-LAP, *S*-SAD_			SAD_*S*-EAR, *S*-DES_	
	λ	λ	λ_*G*_	λ_*S*_	λ_*G*-SAD_	λ_*S*_	λ_*G*-LAP_	λ_*S*_	λ_*G*-CON_	λ_*S*_		λ_*G*-SAD_	λ_*S*_
SAD	1.00 (.91)	1.00 (.90)	1.00 (.86)	1.00 (.32^NS^)	1.00 (.90)		0.48 (.45)	1.00 (.79)	0.78 (.66)	1.00 (.62)	SAD	1.00 (.90)	
SAD	1.05 (.95)	1.07 (.96)	1.11 (.96)	0.34^NS^ (.11^NS^)	1.07 (.96)		0.58 (.54)	1.01 (.79)	0.84 (.71)	1.03 (.64)	SAD	1.05 (.95)	
SAD	1.02 (.93)	1.03 (.92)	1.04 (.89)	0.66^NS^ (.21^NS^)	1.03 (.92)		0.51 (.47)	1.01 (.80)	0.80 (.68)	1.01 (.63)	SAD	1.02 (.92)	
LAP		1.00 (.92)	0.61 (.53)	1.00 (.75)	0.57 (.52)	1.00 (.75)	1.00 (.92)		0.49 (.41)	1.00 (.82)	EAR	0.34 (.31)	1.00 (.87)
LAP		0.98 (.91)	0.55 (.47)	1.04 (.78)	0.52 (.46)	1.04 (.78)	0.98 (.91)		0.41 (.35)	1.03 (.84)	EAR	0.36 (.32)	1.04 (.90)
LAP		0.98 (.91)	0.54 (.46)	1.07 (.79)	0.50 (.45)	1.06 (.80)	0.98 (.91)		0.40 (.34)	1.05 (.86)	EAR	0.40 (.36)	0.92 (.80)
CON		1.00 (.85)	0.70 (.61)	1.00 (.61)	0.66 (.59)	1.00 (.62)	0.31 (.29)	1.00 (.81)	1.00 (.85)		DES	0.77 (.70)	1.00 (.59)
CON		1.13 (.96)	0.83 (.71)	1.07 (.65)	0.78 (.70)	1.08 (.67)	0.41 (.38)	1.08 (.88)	1.13 (.96)		DES	0.70 (.63)	1.00 (.59)
CON		1.02 (.86)	0.80 (.69)	0.80 (.49)	0.75 (.67)	0.81 (.51)	0.43 (.40)	0.93 (.75)	1.02 (.86)		DES	0.79 (.72)	1.07 (.63)
Variance (standard deviation)													
G			0.74 (1)										
SAD	0.82 (1)	0.81 (1)	0.10^NS^ (1)		0.81 (1)		0.62 (1)		0.38 (1)		SAD	0.82 (1)	
LAP		0.85 (1)	0.56 (1)		0.57 (1)		0.85 (1)		0.67 (1)		EAR	0.75 (1)	
CON		0.72 (1)	0.37 (1)		0.39 (1)		0.66 (1)		0.72 (1)		DES	0.35 (1)	
Covariance (correlation)													
SAD, LAP		0.43 (.52)	^F0^		^F0^		^F0^		0.18 (.37)		EAR, DES	0.04 ^NS^ (.07 ^NS^)	
SAD, CON		0.56 (.74)	^F0^		^F0^		0.43 (.67)		^F0^				
LAP, CON		0.31 (.40)	^F0^		0.01 ^NS^ (.02 ^NS^)		^F0^		^F0^				

*Note.* Standardized (in brackets) and unstandardized model parameters for the single factor model (SFM), the correlated factor model (CFM), the symmetrical bifactor model (BF_SYM_), and the bifactor-(*S*−1) models (BF_*S*−1_) with different reference domains. All estimates are significant with *p* < .001, if not indicated otherwise. Facets: LAP = low appetite; CON = concentration problems; SAD = sadness; EAR = early awakening; DES = problems in decision-making; λ = factor loading on a first-order factor; λ_*G*_ = loading on the general factor; λ_*S*_ = loading on the specific factor; ^NS^ = not significant; ^F0^ = parameter fixed to zero. Standard errors are provided online: https://osf.io/sq4zd/

#### Anomalous Results

In the BF_SYM_ model, the specific sadness factor collapsed. Standardized factor loadings of indicators assessing sadness were small and nonsignificant (standardized loadings: λ_SAD1_ = .32, *SE* = .17, *p* = .062, λ_SAD2_ = .11, *SE* = .25, *p* = .661, λ_SAD3_ = .21, *SE* = .27, *p* = .436). Factor variance of the specific sadness factor was also very small (.10, *SE* = .11). Therefore, the BF_SYM_ model became an empirical BF_*S*−1_ model with uncorrelated specific factors in which items assessing sadness defined the meaning of the general factor (*G*_SAD_). As is typical in empirical applications, we removed the collapsing specific factor for sadness. The resulting BF_*S*−1_ model with sadness as a reference domain fit the data well (see Table 1). We allowed the *S* factors of the BF_*S*−1_ model to correlate because, as described above, constraining these correlations would be unnecessarily restrictive. In our example, the partial correlation between the specific concentration factor and the specific appetite factor was close to zero (.02), indicating that concentration problems and low appetite have nothing in common after sadness is partialled out. In order to illustrate that the sadness factor is in fact the same across models, we also constructed a one-factor model using only the three indicators assessing sadness.

Results are summarized in Table 2 and support the idea that the general factor in our BF_SYM_ model is a latent variable underlying the indicators of the collapsing factor. Standardized factor loadings of indicators assessing sadness are very similar across models. In addition, the factor scores of the general factor in the original BF_SYM_ model before any *S* factor was dropped correlated almost perfectly with (a) factor scores of the reference factor in the BF_*S*−1_ model with sadness as reference domain and correlated *S* factors (.99), (b) factor scores of the sadness factor in the model with correlated factors (.99), and (3) factor scores in the single-factor sadness model (.98). Moreover, model-implied correlations between the three SAD indicators observed in the BF_SYM_ (.86, .84, and .88) are also similar to those of (a) the BF_*S*−1_ model with sadness as reference domain (.86, .83, and .88), (b) the correlated factor model (.86, .83, and .88), and (c) the single factor sadness model (.86, .84, and .88).

#### Correlation With Stress and Sleep Quality

Although we tried to estimate correlations of latent variables with stress and sleep quality, the B_SYM_ model did not converge, and standard errors could not be computed. To examine relations between the general factor and external variables, we, therefore, used factor scores from the previous models in which stress and sleep quality were not considered. Correlations of factor scores of the general factor in the original BF_SYM_ model (before the specific sadness was a factor was dropped) with stress (.42) and sleep quality (−.54) were virtually identical to correlations of the general factor in the BF_*S*−1_ model with sadness as reference and correlated specific factors (.41 and −.54, respectively). The same was true for correlations of the sadness factor from the model with correlated factors (.41 and −.54, respectively) and the single-factor model that included only sadness (.40 and −.52, respectively).

#### Summary

High correlations between factor scores from the different models and near-identical correlations with stress and sleep quality support the assertion that when an *S* factor collapses, the general factor in the B_SYM_ model becomes the latent variable underlying the indicators with the non-significant or very small *S* factor loadings. Researchers do not obtain the intended “overarching” factor, but instead model a general factor *G*_i_, which is defined empirically by a specific domain of psychopathology.

### Consequences of Omitting Different *S* Factors

In the first part of the illustrative example, we removed the specific sadness factor because indicators of that factor showed anomalous results. This decision was purely data-driven, as is typical in most applications of bifactor modeling. We now illustrate that we could have removed any other *S* factor instead to avoid anomalous results. However, this results in different meanings of the general factor and remaining *S* factors. To illustrate, we additionally estimated a BF_*S*−1_ model with low appetite as reference and a BF_*S*−1_ model with concentration problems as reference. We also examined how the interpretation of correlations changes when different *S* factors are omitted.

#### Model Fit

Regardless of which *S* factor was removed, all models fit the data well (see Table 1).

#### Factor Loadings

All BF_*S*−1_ models provided reasonable parameter estimates. No anomalous results were encountered (see Table 2). Items that defined the reference domain in each BF_*S*−1_ model are highlighted in color. Several points should be mentioned. First, the (un-) standardized factor loadings of indicators of the reference domain are equal in the BF_*S*−1_ model and the model with correlated factors. Second, the variance of the latent variable representing the general factor in the BF_*S*−1_ model is the same as the variance of the corresponding factor in the correlated factors model. This set of findings indicates that the latent variables are the same. If the specific sadness factor is removed, the general factor reflects sadness. If the specific concentration problems factor is removed, the general factor reflects concentration problems. If the items that measure low appetite are used as a reference, the general factor reflects low appetite.

#### Correlations Between S Factors

Correlations between *S* factors change depending on which domain is used as reference. Using low appetite as reference, the partial correlation between the specific sadness and concentration factors is large (.67).^1^ In contrast, the correlation between the specific concentration factor and the specific low appetite factor in a BF_*S*−1_ model with sadness as reference (.02), and the correlation between the specific appetite factor and the specific sadness factor in a BF_*S*−1_ model with concentration problems as reference (.37) are lower. These differences are to be expected, as the size of correlations between factors in the first-order correlated factors model also varies.

#### Correlation With Stress and Sleep Quality

Correlations of sleep quality and stress with latent variables are summarized in Table 3. A pattern is apparent: Correlations with the general factor always correspond to correlations with the factor from the model with correlated factors if this factor is the one used as reference domain in the BF_*S*−1_ model. For example, the correlation between sadness and stress in the model with correlated factors is moderately high (.43) and, most importantly, of the same size as the correlation between the general factor and stress in the BF_*S*−1_ model with sadness as reference (.43). Correlations with the general factor, therefore, represent correlations with the reference domain. Correlations between the *S* factors and external variables change depending on which reference domain is used. This is to be expected; these correlations are semi-partial correlations, as only the *S* factors and not the external variables are controlled for the influence of the reference domain (this reasoning also applies to Caspi et al., 2014).

**Table 3.** Covariances and Correlations of Stress and Sleep Quality with the Latent Variables.

Model	Stress at work			Sleep quality		
	SAD	LAP	CON	SAD	LAP	CON
CFM _SAD, LAP, CON_	0.38 (.43)	0.19 (.20)	0.37 (.44)	−1.14 (−.55)	−0.90 (−.42)	−0.94 (−.48)
BF_*S*−1_, _SAD, *S*-LAP, *S*-CON_	0.38 (.43)	−0.02 (−.03)	0.12 (.19)	−1.14 (−.55)	−0.28 (−.16)	−0.17 (−.12)
BF_*S*−1_, _LAP, *S*-SAD, *S*-CON_	0.29 (.38)	0.19 (.20)	0.32 (.39)	−0.70 (−.39)	−0.90 (−.42)	−0.64 (−.34)
BF_*S*−1_, _CON, *S*-SAD, *S*-LAP_	0.09 (.15)	0.02 (.03)	0.37 (.44)	−0.42 (−.29)	−0.47 (−.25)	−0.94 (−.48)

*Note.* SAD = sadness; LAP = low appetite; CON = concentration problems; CFM = correlated factor model; BF_*S*−1_ = bifactor-(*S*−1) model. Standard errors are provided online: https://osf.io/sq4zd/

#### Summary

To summarize, we illustrated that no matter which *S* factor is removed from the BF_*S*−1_ model, anomalous results are avoided. In addition, even though the meaning of the general factor changes depending on which domain serves as reference (i.e., which *S* factor is omitted), the meaning of the general factor is clear in each version of the model: The general factor in this approach is the common factor that pertains to the reference domain. This underlines that the decision regarding which specific factor to remove should not be data-driven but should be based on a priori theoretical or substantive reasons. In addition, the general factor should not be interpreted as a general factor of psychopathology unless items pertaining to the reference domain represent direct measures of general psychopathology.

### Consequences of Exchanging *S* Factors in BF_*S*__−__1_ Models

Last, we show that as long as the reference domain remains the same in BF_*S*−1_ models, the meaning of the general factor is constant across applications that use different nonreference facets. For this illustration, we estimate an additional BF_*S*−1_ model that includes domains of early awakening and decision problems instead of low appetite and problems with decision-making.

#### Model Fit

Fit of all estimated models is summarized in Table 1. All models fit the data reasonably well.

#### Factor Loadings and Variances

Factor loadings of the three indicators assessing the reference factor (sadness) are very similar in both models (see Table 2). The same applies to variances of the factors (.81 and .82, respectively). Correlations between factor scores of the general factors are high (.99), indicating that the latent variables are the same, even though the entire set of *S* factors was exchanged.

#### Correlation With External Variables

Increased sadness is associated in the same way with decreased self-rated sleep quality (−.55) and increased impairment due to stress at work (.43) in both BF_*S*−1_ models.

### Overall Summary of the Illustrative Example

We showed that the general factor in a BF_SYM_ model with a collapsing *S* factor is the same as (a) the corresponding first-order factor from the model with correlated factors, (b) the general factor of a BF_*S*−1_ model in which the collapsing factor is considered as reference, and (c) a single-factor model that comprises only the indicators of the vanishing *S* factor. Thus, regardless of whether (a) a reference domain is defined a priori (e.g., Burns et al., 2020a; Heinrich et al., 2020; Junghänel et al., 2020), (b) *S* factors or *S* factor loadings are removed based on empirical results (e.g., Caspi et al., 2014; Tackett et al., 2013), or (c) nonsignificant factor loadings lead to the reduction of a BF_SYM_ model to an empirical BF_*S*−1_ model (e.g., Castellanos-Ryan et al., 2016; Gluschkoff et al., 2019; Martel et al., 2017), consequences for changing interpretation of the general factor and *S* factors are the same: The general factor is no longer interpretable as an “overarching” factor but instead carries a meaning defined by a specific set of symptoms/domains, and the *S* factors are contrasted against that factor.

To avoid a sample-specific, data-driven result regarding what the general factor measures, researchers should define the general factor *a priori* using the BF_*S*−1_ approach. When doing so, the meaning of the general factor remains the same, whether domains are added or removed. The BF_*S*−1_ model also allows for meaningful correlations between specific factors that represent partial correlations among domains after the reference domain has been partially out.

## Discussion

Bifactor models are often used to investigate the latent structure of psychopathology. In this manuscript, we argue that BF_SYM_ models are of limited use for modeling *P* because the interpretation of the general factor is typically ambiguous and lacks comparability across studies. We presented the BF_*S*−1_ approach as a reasonable alternative that (a) guides interpretation of anomalous results in BF_SYM_ models, (b) avoids anomalous results in empirical applications, and (c) assigns each factor a well-defined, theory-based meaning and interpretation. However, researchers who apply the BF_*S*−1_ approach must hypothesize and define a priori which construct they consider transdiagnostically or theoretically meaningful.

### Symmetrical Bifactor Models Are of Limited Usefulness

Consistent with work by others, we point out that the current practice of BF_SYM_ modeling is problematic when analyzing multi-faceted clinical constructs (Bonifay et al., 2017; Burns et al., 2020a; Eid et al., 2017, 2018; Heinrich et al., 2020; Levin-Aspenson et al., 2021; Sellbom & Tellegen, 2019; van Bork et al., 2017; Watts et al., 2019, 2020). From a statistical point of view, it is possible to add a general factor to *any* model that consists of several correlated first-order factors. BF_SYM_ models almost always lead to improved model fit because they can mask minor misspecifications (e.g., Geiser et al., 2015; Greene et al., 2019; Murray & Johnson, 2013) and have a better propensity to fit arbitrary data patterns compared with competing factor models—even if the number of free parameters is the same (Bonifay & Cai, 2017). An often underappreciated but important question is whether including a general factor leads to a more meaningful and interpretable model of psychopathology. If researchers use BF_SYM_ models in conjunction with structurally different domains, we believe the answer to this question is clearly “no.” Structurally different domains often have heterogeneous patterns of correlations. These heterogeneous correlations can cause inconsistent factor loadings and factor collapse in BF_SYM_ models, leading to the general factor turning into a domain- or even symptom-specific factor.

Even in instances in which no anomalous results occur, we consider BF_SYM_ models inappropriate for characterizing the multi-faceted structure of psychopathology. For example, anomalous results are less likely if correlations between structurally different domains are similar and artificially “mimic” interchangeability. However, whether domains are structurally different is not an empirical determination but rather a conceptual one. Thus, empirical results provide at best an indication of how plausible the interchangeability assumption is. From this standpoint, *application of a BF_SYM_ model to structurally different domains always leads to a general factor without clear meaning, even if a solution contains all admissible parameter estimates*. We do not know what this factor means beyond the fact that it accounts for correlations among different domains. This ambiguity cannot be resolved by relating *P* to external variables. Using a BF_SYM_ model, it remains unclear whether an association with the general factor is an association with general psychopathology or with something completely different. The same is true when the general factor is used as a predictor in a regression model. Does general psychopathology, a specific psychopathology, or something quite different explain variance in the dependent variable? None of these questions can be answered conclusively, which limits the value of testing substantive hypotheses about mechanisms underlying various mental disorders.

In addition, the meaning of *P* in BF_SYM_ models is likely not invariant across studies that use different psychopathologies as domains and/or in which different symptoms drop their *S* factor. Consider the previously cited *P* studies. In Lahey et al. (2012), *P* would be most appropriately described as a GA/AP factor, whereas in Caspi et al. (2014), Romer et al. (2021), and Laceulle et al. (2015), *P* represented TD. In Tackett et al. (2013) and Watts et al. (2019), *P* was defined by both generalized anxiety and major depression. In Brandes et al. (2019), it was defined by two items assessing depressive mood. In Swales et al. (2020), *P* represented a latent variable underlying attention/social/thought problems, and in Snyder et al. (2017), the general factor was defined by two indicators assessing hyperactivity and inattention. In Martel et al. (2017), it captured autism in children and separation anxiety in mothers. Thus, the meaning of *P* varies from study to study. Naming all general factors as the “general factor of psychopathology” or “*P* factor” misleadingly suggests a consistency where none exists. Levin-Aspenson et al. (2021) underscored that even *P* factor models using the same set of indicators can yield general factors with substantially different interpretations across samples. In this context, the common interpretation of *P* as a “unifying” dimension across samples and forms of psychopathology will almost certainly lead the field down blind alleys and toward false conclusions about the nature of mental illness.

Indeed, correlations of *P* with external variables are not comparable across studies, making a meaningful accumulation of scientific knowledge in systematic reviews and meta-analysis difficult if not impossible. For example, the correlation of neuroticism with *P* found by Brandes et al. (2019) was almost twice as large (.81 vs. .43) as the correlation reported by Caspi et al. (2014). However, in Brandes et al. (2019) the *n*-factor *and* the *P* factor both represented depressed mood, whereas, for Caspi et al. (2014), *P* depicted TD. Although these findings may show that neuroticism is more strongly associated with depressed mood than with TD, they do not yield a unifying *P*, much less showing how strongly any such *P* is associated with neuroticism.

The fact that almost every *P* factor study uses a different set of psychological assessments poses additional problems. Simply finding a general factor in datasets that assess different domains of psychopathology with completely different assessments does not mean that *the* general factor of psychopathology has been identified or replicated. Different researchers have highlighted that psychological assessments are not interchangeable (e.g., Fried & Nesse, 2015; Østergaard, 2018). Each scale contains specificity due to response format(s) and assessment procedures and because they do not always assess the same symptoms. Even if there were a general factor of psychopathology and this factor could be represented in BF_SYM_ models, it is unlikely that different measures would capture *P* in the same manner. Rather, *P* would carry method-specific meaning. Worse, there is no way to test measurement invariance if *P* is always measured differently.

### Toward Direct and Unambiguous Assessment of *P*

Most studies of *P* seek to identify a *general* factor that is extracted based on the *entire* set of symptoms. In contrast, the BF_*S*−1_ approach does not include a general overarching factor. Instead, it defines the general factor as a factor underlying indicators of the reference domain. This is a very different approach to studying a general psychopathology factor and deviates from the common practice of modeling a general factor and trying to figure out what this factor might measure. However, trying to measure the construct assumed to be “transdiagnostically meaningful” directly seems appealing, considering that researchers who use BF_SYM_ models usually do not find/depict the intended general *P* factor either, as we have demonstrated in this article. The need for more theory-based approaches to psychological constructs underlying various forms of psychopathology was recently underscored by Levin-Aspenson et al. (2021). The authors argued that “future studies would do well to examine the *P* factor against hypotheses about its nature (e.g., cognitive and/or emotional dysregulation, dynamic developmental processes) [. . .]” (p. 1,045). Thus, if researchers (a) have a theory about the meaning of the general factor and (b) use a measurement instrument that captures the construct directly based on that meaning, problems such as lack of comparability and interpretational ambiguities are resolved, so meaning becomes transparent and replicable.

Defining, selecting, and measuring the appropriate BF_*S*−1_ reference facet are challenging tasks. As described above, the reference facet defines the meaning of the general factor, and changing the reference facet means changing the meaning of the general and specific factors. More importantly, general factors of BF_*S*−1_ models that use noninterchangeable reference facets are not comparable across studies. Despite these characteristics of the BF_*S*−1_ approach, choosing the reference facet *a priori* is probably no more complex than trying to figure out *post hoc* what the general factor in a BF_SYM_ model means. The BF_*S*−1_ approach is complex in a different way because it motivates researchers to develop theories about the construct they consider transdiagnostically or developmentally meaningful.

As has been emphasized elsewhere, BF_*S*−1_ models are not a panacea for all issues related to modeling psychopathology (Burns et al., 2020b). BF_*S*−1_ models address the questions that BF_SYM_ models try to answer (“*What underlies various domains of psychopathology*”) with the advantageous property that the search for meaning is not data-driven, but that meaning is ensured by careful selection of indicators for the reference facet (Burns et al., 2020b). Of course, it can be argued that BF_*S*−1_ models are not a sensible approach for *P* factor research. This reservation can be justified if researchers conceptualize *P* in a way that is not compatible with the BF_*S*−1_ approach, or if researchers think that *P* cannot be measured directly. In this case, however, one must bear in mind that the same caution should also apply to the many BF_SYM_ models that are reduced to an empirical BF_*S*−1_ model due to collapsing *S*-factors and non-significant *S*-factor loadings. In models that include these kinds of anomalous results, researchers measure the general factor directly with indicators that load exclusively on the general factor—which is typically not intended.

Of note, neuroticism (also referred to as negative affectivity and negative emotionality) appears to be a promising “transdiagnostically meaningful” construct, given links to a wide range of psychopathologies, including both internalizing and externalizing symptoms and their co-occurrence across the life span (Beauchaine & Tackett, 2020; Brandes et al., 2019; Caspi et al., 2014; Olino et al., 2014; Tackett et al., 2013). If a researcher considers neuroticism to be at the core of the meaning of *P*, neuroticism or a highly related construct (negative emotionality, negative affectivity) should be assessed directly. See also Caspi and Moffitt (2018) or Smith et al. (2020) for four possible ways to define general factor consistent with the BF_*S*−1_ approach (i.e., diffuse unpleasant affective state, impulse control over emotions, deficits in certain intellectual functions, disordered thought). These definitions provide guidance in the selection of a suitable measurement instrument for the direct assessment of *G*_i_ and/or for the choice of the reference domain in the BF_*S*−1_ approach.

**Figure 2:**
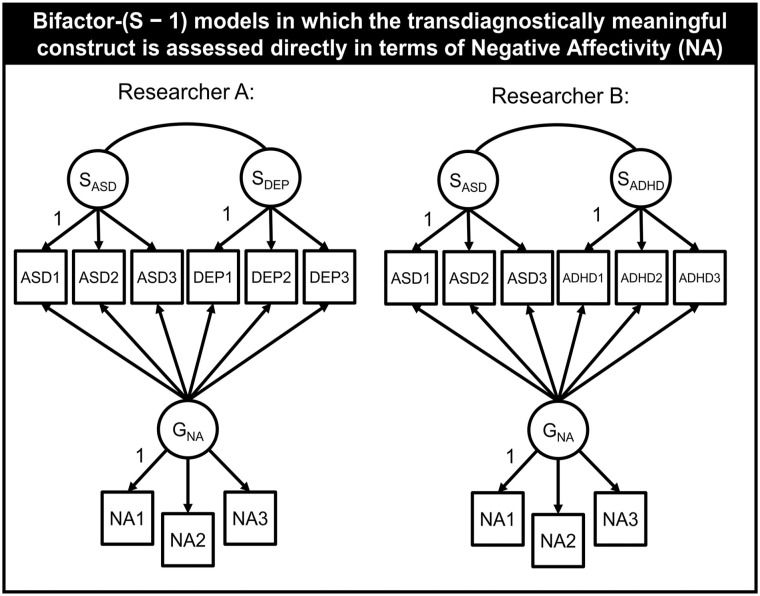
Two Different Bifactor-(S–1) Models With Indicators That Assess Negative Affectivity (NA) as Markers for the Reference Domain *Note.* Thus, the general factors have the same meaning in both models. Although Researcher A is interested in how much variance is shared between autism spectrum disorder (ASD) and depression (DEP) can be explained by NA, Researcher B is interested in how much variance shared between attention-deficit/hyperactivity disorder (ADHD) and ASD can be explained by NA. The partial correlations between the specific factors indicate what both factors have in common once the effect of NA has been partially out. As both researchers use the same reference, they can compare their results and accumulate knowledge.

By assessing *P* directly, one can ensure the general factor carries the same meaning in different studies. See Figure 2 for an illustration in which is negative affect (NA) is treated as reference. Suppose that Researchers A and B consider NA as the defining domain for the general factor and use NA as a marker for the reference domain. Researcher A is interested in the relationship between *G*_NA_, autism, and depression. Therefore, she represents both psychopathologies as *S* factors. Researcher B proceeds in the same way, with the difference that he models *S* factors for autism and ADHD. By using the same reference domain (NA), assessed with the same measurement instrument, both researchers give the general factor the same meaning, allowing them to accumulate knowledge and compare findings (see Figure 2).

### Recommendations for Selecting an Appropriate Bifactor Model

**Figure 3:**
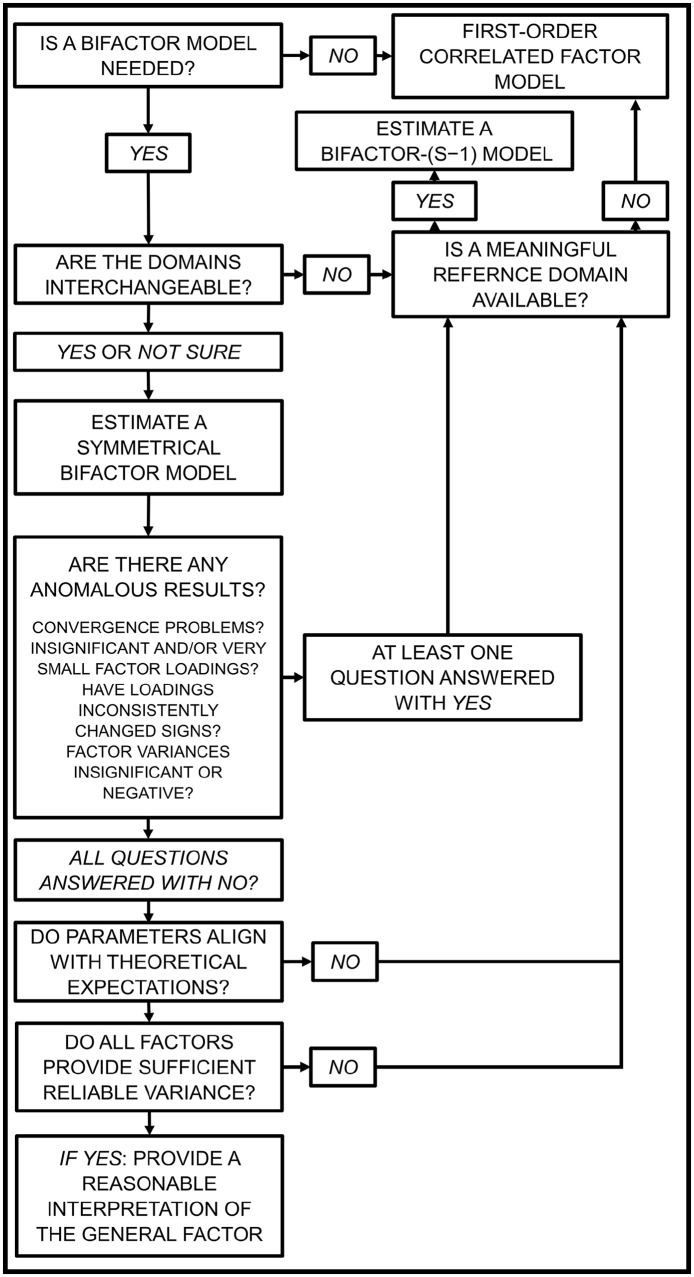
Decision Tree to Decide Between Different Variants of Bifactor Structures

Based on the arguments outlined in this manuscript, we present a decision tree to help researchers choose a suitable bifactor modeling approach (Figure 3). These recommendations transfer ideas developed in multi-method measurement approaches (Eid, 2000; Eid & Koch, 2014; Geiser et al., 2012) to bifactor structures used to model psychopathology and to all contexts in which bifactor models are used. We are aware that not everyone shares our critical view of BF_SYM_ models and considers the interchangeability of domains as a necessary prerequisite; we have tried to take that into account. Next, we will briefly describe each step of the decision tree, which is based on the assumption that researchers can access well-defined facet factors with clear meanings assessed with items that approach the ideal of reflective indicators.

Initially, one must decide whether a bifactor structure is necessary to answer a specific research question, why a bifactor model is the most appropriate modeling framework, and, in particular, why a bifactor model is more appropriate than a simpler correlated factors model. From a substantive perspective (which is widely used in *P* factor research), the initial assessment should consider whether bifactor structure is theoretically defensible, that is, whether a “transdiagnostically meaningful” construct is associated with any form of psychopathology exists and can be depicted appropriately in a bifactor model. Elaborated discussions on this issue have been published elsewhere and are beyond the scope of the current manuscript (e.g., Aristodemou & Fried, 2020; Bonifay & Cai, 2017; Smith et al., 2020; van Bork et al., 2017; Watts et al., 2020). From a measurement perspective, researchers should ask whether the bifactor structure can summarize covariances among manifest indicators of psychopathology in a way that is readily interpretable and comparable across studies.

In any case, researchers should explicitly define (a) what they mean by “transdiagnostically meaningful” and (b) how this understanding translates into estimated model parameters (e.g., Does “transdiagnostically meaningful” mean a large proportion of explained variance in all or a subset of the non-reference facets? Do all indicators need to load on the general factor and a specific factor? Should the general factor explain all covariances among nonreference facets? Is it conceptually problematic if indicators change factor loadings inconsistently?), (3) and how the bifactor model at hand ensures interpretability and comparability with existing research. Regardless of whether the initial assessment is for or against using a bifactor structure, using a bifactor model always means giving up the clarity of a model with correlated factors. Therefore, we recommend that correlated factors models always be analyzed and reported as a first step because these models provide useful context to understand findings from bifactor models (Eid, 2020).

If a bifactor model is required, one must determine whether domains can be considered interchangeable or not. If the answer is “no, the domains are structurally different,” we recommend choosing a suitable reference domain and estimate a BF_*S*−1_ model. We argue that domains in clinical psychology are rarely, if ever, interchangeable and that correlated factor models or BF_*S*−1_ models are most useful for the vast majority of research questions. However, as discussed above, it is important to keep in mind that choices of reference domains should be made based on theory and substantive considerations rather than empirical model fit (Eid, 2000, 2020; Geiser et al., 2008; Heinrich et al., 2020). As discussed above, such a theory-based decision is necessary to receive well-defined and interpretable general and specific factors, and to avoid comparability and replicability problems due to data-driven selection of reference facets. If no theoretically sound reference facet is available, we recommend using the first-order correlated factors model.

If the answer to the question of whether domains are interchangeable is “yes, domains are interchangeable” or “I am not sure,” then estimating a BF_SYM_ model is a tenable first step. Nonconvergence may indicate that the model is inappropriate. If the model converges, estimated parameters should be examined critically. Are there insignificant factor loadings? Are there factor loadings with altered signs compared with the first-order model with correlated factors? Are there factors with a nonsignificant or negative variance estimate? If the answer to any of these questions is “yes,” the domains may be better viewed as structurally different. In that case, we would use the BF_*S*−1_ model, and, if a meaningful reference domain is not available, a correlated factors model.

Note that anomalous results are not necessarily limited to a single facet. For example, multiple facets may collapse. This may occur if models include multiple statistically indistinguishable (i.e., highly correlated) facets. In addition, it is also possible that only a subset of indicators from one facet or subsets of indicators from different facets collapse into the general factor. The latter types of anomalous results may be related to the homogeneity of the indicators and to which facets the indicators are assigned.

If indicators are homogeneous, the whole facet should collapse because homogeneous indicators should behave similarly. If indicators within a particular facet form homogeneous subsets, we expect only one homogeneous subset to collapse into the general factor. When indicators from different facets collapse into the general factor, the collapsing indicators may have more in common than indicators within the same facet. In any case, investigators should ask about possible reasons for the anomalous findings.

Explanations for unexpected loading patterns may be found in item content. For example, in Castellanos-Ryan et al. (2016), the specific externalizing factor is partially collapsed. Although the indicators *drinking problems*, *drug use*, and *smoking frequency* showed significant *S* factor loadings, indicators assessing *ADHD*, *conduct disorder*, and *ODD* of the same *S* factor did not. ADHD-related items may form a homogeneous subset under the externalizing factor and behave similarly (and thus collapsed into the general factor). Anomalous results should always result in a careful re-evaluation of the measurement model.

It is important to consider that additional parameter constraints and Bayesian estimation methods, and large samples can stabilize the BF_SYM_ model and help avoid inadmissible parameter estimates. Such models converge, and at first glance, appear appropriate, even when interpretational problems remain. In such situations, further evidence that the BF_SYM_ bifactor model is reasonable should be provided. We recommend that researchers (a) explore whether the loading patterns align with theoretical expectations and (b) whether factors depict a sufficient amount of reliable variance. Both considerations are linked to suggestions made by Watts et al. (2019) and offer a strategy to decide for or against a BF_SYM_ model. We briefly summarize the proposed evaluations and describe what can be expected when a BF_*S*−1_ model is used (for a comprehensive discussion, see Watts et al., 2019).

First, researchers should explore whether model parameters (e.g., all factor loadings and correlations between specific factors) align with theoretical expectations. These expectations and corresponding empirical results should be reported and discussed. For example, Watts et al. (2019) argue that the effects of the general factor on all indicators of all *S* factors should be roughly the same, which is in line with the idea of interchangeable facets (Eid, 2020). This consideration is vital for BF_SYM_ models, as researchers are forced to make the expected relation between the general factor and indicators transparent and test these expectations. This, however, is rarely done in practice.

When a BF_*S*−1_ model is used, it is not necessary for all items to be equally linked to the general factor. Consider our empirical example, in which the correlation between sadness and concentration problems was stronger than the correlation between sadness and low appetite. Consequently, when sadness is used as a reference, indicators of concentration problems will show higher loadings on the general factor than indicators of low appetite (see Table 2). That is, the size of factor loadings of the items of the nonreference domains can vary across domains without indicating any problems in the BF_*S*−1_ approach.

Second, Watts et al. (2019) suggest that each *S* factor should reflect an appropriate amount of reliable variance. This is consistent with other authors, who have argued that collapsing *S* factors are problematic (Eid, 2000; Geiser et al., 2015). That test is particularly useful when the sample is large, where even small loadings are significant. Low variance in one of the *S* factors indicates problems. For the BF_*S*−1_ model, however, the situation is different. In contrast to BF_SYM_ models, low specificity does not provide evidence that the model should be rejected. Consider a factor model that includes the two highly correlated factors sadness and pessimism (*r* = .80, 64% shared variance). Using sadness as a reference, the specific pessimism factor represents that part of pessimism that cannot be predicted by sadness, that is, what is unique after sadness is considered. Given shared features of sadness and pessimism, only a small amount of residual variance remains.

But even if no anomalous results occur and a BF_SYM_ model passes these tests, it is still unclear what the general factor in a BF_SYM_ model measures because it is not defined by a specific set of indicators. To infer its meaning, researchers typically estimate correlations with external clinical variables and sometimes compare these correlations across studies. However, such comparisons are only meaningful when two bifactor models have the same structure. Proper consideration of comparability is particularly important if different measurement instruments are used. Because direct tests of measurement invariance are often not feasible, we recommend the following. First, researchers should investigate whether the models are specified in a similar manner (e.g., whether correlations between the *S* factors are allowed). Second, they should check whether both models contain the same set of domains and whether these domains are measured using the same indicators. And third, they should verify whether patterns of factor loadings are comparable. If two models differ in any of these aspects, it is unlikely the general factor has the same meaning, or that correlations are comparable across studies. This limitation of comparability should be explicitly pointed out.

### Limitations

Some limitations of the present work should be considered. First, our arguments focus exclusively on bifactor models. Interchangeability is also a necessary prerequisite for meaningful interpretation of higher order factors in hierarchical factor analytic models (Eid et al., 2017). Second, we focus on interchangeability of domains as a critical feature to decide for or against specific variants of bifactor models. Of note, the meaning of bifactor models has also been scrutinized from perspectives other than those represented in the manuscript (e.g., van Bork et al., 2017). Third, we focus on modeling the general factor of psychopathology, but the same arguments apply to other applications aiming at modeling disorder-specific general factors, including general factors of depression, anxiety, ADHD, and all other mental disorders (e.g., Burns et al., 2020a; Heinrich et al., 2020; Junghänel et al., 2020). Fourth, we focus exclusively on the bifactor approach. Several other modeling approaches, such as formative measurement models and network models, have been proposed (see Caspi & Moffitt, 2018, for an overview). Fifth, all arguments presented above assume that indicators are homogeneous within their domain. Exemplarily, they assume that symptoms of mania, psychosis, and OCD are unidimensional indicators of the latent variable thought disorder. Whether this assumption is reasonable for TD and other symptom domains is beyond the scope of this manuscript.

## Conclusion

The widespread use of bifactor approaches to model the general factor of psychopathology must be viewed critically. Researchers who use BF_SYM_ models often encounter high levels of ambiguity in the meaning of the modeled general factor. In most cases, these factors do not represent general psychopathology but rather a specific domain of psychopathology. BF_*S*−1_ models are a useful alternative that circumvents many problems of BF_SYM_ models. With this approach, the general factor has a clear meaning defined by the reference domain—though this factor should not be interpreted as the general factor of psychopathology unless *P* is assessed directly. The BF_*S*−1_ approach allows for meaningful comparisons of *P* across different informants, samples, time points, and studies. We hope that this manuscript will help researchers decide for or against the use of a bifactor structure, and to assess whether the use of a BF_SYM_ model is truly the most fruitful approach to studying what different mental disorders have in common.

## Supplemental Material

sj-docx-1-asm-10.1177_10731911211060298 – Supplemental material for On the Meaning of the “P Factor” in Symmetrical Bifactor Models of Psychopathology: Recommendations for Future Research From the Bifactor-(S−1) Perspective Click here for additional data file. Supplemental material, sj-docx-1-asm-10.1177_10731911211060298 for On the Meaning of the “P Factor” in Symmetrical Bifactor Models of Psychopathology: Recommendations for Future Research From the Bifactor-(S−1) Perspective by Manuel Heinrich, Christian Geiser, Pavle Zagorscak, G. Leonard Burns, Johannes Bohn, Stephen P. Becker, Michael Eid, Theodore P. Beauchaine and Christine Knaevelsrud in Assessment

## Notes

1. It should be noted that the size of the partial correlations can be determined by hand, by simply using the factor intercorrelations of the model with correlated factors and the formula for partial correlations. For example, in the correlated factor model, the correlation between sadness and low appetite is .52, between sadness and concentration problems is .74, and between low appetite and concentration problems .40. Using the formula for partial correlations and computing the partial correlation between sadness and concentration problems while treating low appetite as reference, we obtain

$\begin{array}{l} r_{\left(SAD,\;CON\right)\cdot LAP}=\frac{\left(r_{SAD,\;CON}-r_{LAP,SAD}\times r_{LAP,CON}\right)}{\sqrt{\left[\left(1-r_{LAP,\;SAD}^{2}\right)\times \left(1-r_{LAP,\;CON}^{2}\right)\right]}}\\ \;\;\;\;\;\;\;\;\;\;\;\;\;\;\;\;\;\;\;\;\;\;\;\;\;\;=\frac{.74-.52\times .40}{\sqrt{\left(1-.52^{2}\right)\times \left(1-.40^{2}\right)}}=.68 \end{array}$

which corresponds well to the correlation between specific factors observed in the BF_*S*−1_ model (.67). This again highlights that the nonreference domains are in contrast with the reference factor and that the general factor carries the meaning of the reference domain.
