# Supplementary material for: On the Meaning of the “P Factor” in Symmetrical Bifactor Models of Psychopathology: Recommendations for Future Research From the Bifactor-(S−1) Perspective
Source: Assessment. 2021 Dec 3;30(3):487–507. doi: 10.1177/10731911211060298 (PMC9999288; doi:10.1177/10731911211060298)
Supplement: sj-docx-1-asm-10.1177_10731911211060298 – Supplemental material for On the Meaning of the “P Factor” in Symmetrical Bifactor Models of Psychopathology: Recommendations for Future Research From the Bifactor-(S−1) Perspective [file sj-docx-1-asm-10.1177_10731911211060298.docx]

**Supplemental Material 1**

In the manuscript, we argue that the thought disorder factor in the correlated factor model and the general factor (i.e., the *P* factor) in the Caspi et al. (2014) bifactor model are the same latent variables. This equivalence is not apparent at first glance. This supplementary material illustrates that both latent variables are indeed the same. To reach this goal, we show that BF*_S_*_–1_ models are reformulations of latent regression models (e.g., Burns et al., 2020; Eid et al., 2017; Geiser et al., 2008, 2012; Heinrich et al., 2020; Koch et al., 2018).

We show that the latent regression model and the BF*_S_*_–1_ model imply the same (co)variances for the reference facet indicators and explain that differences in model fit are due to how the non-reference indicators are linked to the reference facet. This linkage is more restrictive in the latent regression model (i.e., implies a proportionality constraint, LR-CON) than in the unconstraint BF*_S_*_–1_ model (i.e., implies no proportionality constraint, BF*_S_*_–1_-UNC). We demonstrate which parameter constraints must be added to the BF*_S_*_–1_ model to obtain the constraint BF*_S_*_–1_ model (BF*_S_*_–1_-CON), which implies the same (co)variances as the LR-CON. Moreover, we present a less restrictive latent regression model that resembles the BF*_S_*_–1_-UNC.

We present all models in a general form. Thus, necessary constraints for model identification must be added (e.g., fixing the first indicator to 1 and latent means to zero).

**Correlated Factor-Model and Constraint Latent Regression Models (LR-CON)**

In a correlated factor model, all manifest indicators $Y_{ij} (i=1, \ldots,I;$*I* = number of indicators *i* belonging to facet *j*) load on a first-order facet factor $T_{j}$ $(j=1, \ldots, J;$*J* = total number of facets) which depicts the latent variables underlying a set of indicators belonging to facet *j*. The following measurement equation is employed:

$$\left( 1 \right){Y_{ij}= a}_{ij}+\lambda_{Tij}\cdot T_{j}+\varepsilon_{ij}$$

In this equation, $\lambda_{Tij}$ denotes a factor loading of item *i* on the corresponding first-order factor $T_{j}$, $a_{ij}$ denotes an intercept, and $\varepsilon_{ij}$ denotes measurement error. Typically, different facets can correlate, Cov($T_{j}$, $T_{j´}) \neq0.$To specify a LR-CON model, one can define one facet as the reference facet (the “overarching general factor” *j*) and regress a non-reference facet (*j´*) on the reference facet (see Figure S1.1 Panel A1). The following equations describe the regressive relation between the reference facet *j* and the non-reference facet *j*´:

(2) $Y_{ij}=\alpha_{ij}+ \lambda_{Tij}\cdot T_{j}+\varepsilon_{ij}$ (reference facet *j*)

(3) $Y_{ij´}=\alpha_{ij´}+\lambda_{Tij´}\cdot T_{j´}+\varepsilon_{ij´}$ (non-reference facet *j'*)

(4) $T_{j´}=\alpha_{jj´}+\beta_{jj´}\cdot T_{j}{+ S}_{j´}$ (regression of *j'* on *j*)

As in the correlated factor model, $\lambda_{Tij}$ and $\lambda_{Tij'}$ denote factor loadings, and $\varepsilon_{ij}$ and $\varepsilon_{ij´}$ denote measurement errors. In addition, $\beta_{jj´}$represents the regression slope describing the linear association between the non-reference facet $T_{j´}$ and the reference facet $T_{j}$, and $\alpha_{jj´}$ denotes the intercept of regression between the two latent variables. The latent variable is a $S_{j´}$ regression residual. The residuals represent the part of the non-reference facets variance that the reference facet cannot explain. The regression residuals of different non-reference facets *j*´ and *j*´´ can correlate, Cov($S_{j´}$, $S_{j´´}) \neq0$. This association is a partial covariance corrected for the influence of the reference facet.

An LR-CON model is conceptually similar to a higher-order factor model. However, unlike higher-order factor models, the general factor is measured directly (i.e., the reference facet is measured with multiple indicators). To understand the similarity, consider Figure S.1. Panel A2. The model is the same as the LR-CON model, except that it includes a phantom variable, which we have labeled $G_{j}$. This phantom variable is derived by fixing the loading of $T_{j}$on $G_{j}$ to 1 and the variance of $S_{j}$to zero (i.e., there is *no* specific variance). Thus, all variance of the lower-order factor $T_{j}$is squeezed into the pseudo higher-order factor $G_{j}$. Although this $G_{j}$ looks much more general than $T_{j}$ in the latent regression model, it is still $T_{j}$. This is similar to empirical higher-order factor models in which lower-order factors collapse.

Although the LR-CON model distinguishes between dependent and independent variables – and is in this way conceptually different from the model with correlated first-order factors – the meaning of latent facet factors is the same in both models. Consequently, Caspi et al. (2014) could have regressed internalizing and externalizing on thought disorder and obtained a model that (1) has the same model fit as the correlated factor model and (2) in which the latent variables have the same substantive meaning as in the correlated factor model. In an LR-CON model, the measurement equation for the indicators of the reference facet is still the same as in the correlated factor model (see equation 5). However, inserting equation (3) into (2) results in equation (6) which shows that the reference facet has an *indirec*t effect on indicators of the non-reference facet that flows from the reference facet through the non-reference facet to each non-reference indicator.

(5) $Y_{ij}=\alpha_{ij}+\lambda_{Tij}\cdot T_{j}+\varepsilon_{ij}$ (reference facet)

(6) $Y_{ij´}=\alpha_{ij´}+\lambda_{Tij´}\cdot\alpha_{jj´}+\lambda_{Tij´}\cdot\beta_{jj´}\cdot T_{j}{+ \lambda_{Tij´}\cdot S}_{j´}+\varepsilon_{ij´}$ (non-reference facet)

Table S1.1 shows the (co)variances implied by an LR-CON. Especially the equation describing the decomposition of variances of the non-reference indicators is interesting. The equation shows that the variance of each non-reference indicator is decomposed into (1) one part explained by the reference facet, (2) one part explained by the residual, and (3) one part reflecting measurement error. This decomposition is well known from higher-order and bifactor models. Moreover, the equation shows that the ratio of the variance explained by the reference facet ($T_{j}$) and by the facet-specific residual factor ($S_{j'}$) is the *same* for all indicators within the same non-reference facet.

(7) $\frac{\lambda_{Tij´}^{2} \cdot{\beta_{jj´}^{2}\cdot Var(T}_{j})}{\lambda_{Tij´}^{2} {\cdot Var(S}_{j´})}$ = $\frac{{\beta_{jj´}^{2} \cdot Var(T}_{j})}{Var(S_{j´})}$

This proportionality constraint is more familiar from higher-order factor models in which lower-order factors load on an overarching general factor (e.g., Gignac, 2016; Schmiedek & Li, 2004; Yung et al., 1999). Due to this constraint, we refer to this model as the *constraint* latent regression model (LR-CON). We now show that the *unconstraint* BF*_S_*_–1_ model (BF*_S_*_–1_-UNC) drop this restriction. This explains why BF*_S_*_–1_-UNC models typically fit the data better than correlated factor models or LR-CON models (Geiser et al., 2008).

**The Constraint (BF*_S_*_–1_-CON)** **and Unconstraint Bifactor-(*S*−1) Model (BF*_S_*_–1_-UNC)**

BF*_S_*_–1_ models decompose the variance of indicators similar to the LR-CON models (see Figure S1.1, Panel B). BF*_S_*_–1_ is conceptually similar to symmetrical bifactor models, except that one specific factor is dropped, and the meaning of the general factor is determined by direct measurement of the reference facet. In a BF*_S_*_–_1-UNC, indicators of the reference facet $\left( j \right)$load only on the general factor $G_{j}$ representing the latent variable underlying the reference indicators. Thus, it would be appropriate to label the general factor $T_{j}$ as in the latent regression model. However, to be in line with standard notation, we label it $G_{j}$. The indicators of the non-reference facets load on the general factor $G_{j}$and a specific factor.

(8) $Y_{ij}=\alpha_{ij}+\lambda_{Gij}\cdot G_{j}+\varepsilon_{ij}$ (reference facet)

(9) $Y_{ij´}=\alpha_{ij´}+\lambda_{Gij´}\cdot G_{j}{+ \lambda_{Sij´}\cdot S}_{j´}+\varepsilon_{ij´}$ (non-reference facet)

In these equations, $\lambda_{Gij}$ and $\lambda_{Gij´}$denote factor loadings on the general factor $G_{j}$,$\lambda_{Sij´}$ denotes a factor loading on a non-reference specific factor $S_{j´}$, $\alpha_{ij}$ and $\alpha_{ij´}$ denote intercepts and $\varepsilon_{ij}$ and $\varepsilon_{ij´}$ denote measurement error. The latent variable$S_{j´}$is a regression residual representing the variance in the non-reference facets that the reference facet cannot explain. It has the same conceptual meaning as the residual variable in the latent regression model. *S* factors can correlate and have a mean of zero by definition.

Comparing equations (5) and (8) reveals that the LR-CON model and the BF*_S_*_–1_ model employ the same measurement equations for the reference facet. Consequently, they imply the same variances and covariances for the indicators of the reference facet. However, equations (6) and (9), which describe the relationships between reference facet and non-reference indicators, differ. The proportionality constraint employed in the latent regression is missing in the BF*_S_*_–1_-UNC model. The ratio of variance attributable to the general factor and the specific factor can differ between indicators within a non-reference facet.

(10) $\frac{\lambda_{Gij´}^{2} \cdot Var(G_{j})}{\lambda_{Sij´}^{2} \cdot Var(S_{j´})}$

This makes sense because the BF*_S_*_–1_-UNC model regresses indicators of the non-reference facets directly on the reference facet (i.e., all indicators load on the general factor), and these loadings are estimated freely without any additional constraints (i.e., the effect does *not* flow through a facet factor as in the LR-CON). Consequently, the (co)variances implied by the unconstrained BF*_S_*_–1_-UNC model for the non-reference indicators are not equivalent to those implied by the LR-CON, whereas the (co)variances implied for the reference indicators are. Said differently: The BF*_S_*_–1_-UNC model is a less restrictive LR-CON model.

To constrain the BF*_S_*_–1_ model equivalent to the latent regression model, one must add the missing proportionality constraint. Thus, one has to insert $\lambda_{Gij´}$ = $\lambda_{Sij´}\cdot\beta_{jj´}$, where $\beta_{jj´}$ denotes a non-reference facet specific constant that is conceptually equivalent to $\beta_{jj´}$ in the LR-CON model. Adding the proportionality constraint results in a *constraint* BF*_S_*_–1_ model (BF*_S_*_–1_-CON). This BF*_S_*_–1_-CON implies the same (co)-variances as the latent regression model (see Table S1.1 and Figure S1.1 Panel D). A consequence of this constraint is that the model fit can no longer distinguish BF*_S_*_–1_-CON with different reference facets since they all fit the data equally well.

**Unconstraint Latent Regression Model (LR-UNC)**

Lastly, it is possible to make the LR-CON is less restrictive. The *unconstraint* latent regression model (LR-UNC, see Figure 1, Panel C1) is conceptually similar to a higher-order factor model with direct effects (Yung et al., 1999). As in the latent regression model, the indicators of the reference facet load exclusively on the reference facet (Chen et al., 2006; Yung et al., 1999). The critical difference between the LR-CON and LR-UNC is that the indicators of the non-reference facets load onto the non-reference facet *and* on the reference facet. Thus, the measurement equations for the non-reference indicators are more complicated than in an LR-CON model:

(11) $Y_{ij}=\alpha_{ij}+\lambda_{Tij}\cdot T_{j}+\varepsilon_{ij}$ (reference)

(12) $Y_{ij'}=\alpha_{ij´}+\lambda_{Tij´}\cdot\alpha_{jj´}+\beta_{jij´}^{D}\cdot T_{j}+\lambda_{Tij´}\cdot\beta_{jj´}\cdot T_{j}{+ \lambda_{Tij´}\cdot S}_{j´}+\varepsilon_{ij´}$

(non-reference)

First, and most importantly, the measurement equation for the indicators of the reference facet is the same as in the other models. The difference between equation (8) and equation (12) is $\beta_{jij´}^{D}\cdot T_{j}.$ This product denotes the direct effect of the reference facet on the indicators of the non-reference facets. Conceptually, the proportionality constraint of the LR-CON model is still present ($\lambda_{Tij´}\cdot\beta_{jj´}\cdot T_{j})$ but is overridden by allowing this direct effect. This explains why the LR-UNC provides the same model fit as the BF*_S_*_–1_-UNC. Like the LR-CON model, the LR-CON can be supplemented by a phantom general factor (Figure S1.1 Panel C2). Again, the $G_{j}$ is the same latent variable as $T_{j}.$

**Summary**

This supplemental explained the relation between six variants of conceptually similar latent regression models. Figure S1.1 summarizes these relations. The figure is an adapted version of the Figure presented by Yung et al. (1999), who summarized the relationships between higher-order and bifactor factor models. All models are conceptually similar to these presented by Yung et al. (1999). Our illustration explained that (1) all models imply the same (co)variances for the indicators of the reference facet, and (2) differences are limited to the relation between the reference facet and the non-reference indicators. Although these differences influence fit indices, they are irrelevant for the meaning of the latent variable underlying the indicators of the reference facet, irrespective of whether this is presented as a separate facet in the latent regression models or as a general factor in the BF*_S_*_–1_ model. A researcher may wonder which they should favor. We prefer the BF*_S_*_–1_ model because they represent facet-specific variance explicitly, and thus, it is easier to add predictors and covariates of the facet-specific variance. Note that this is also possible in the other models; however, somewhat more complicated.

**Example**

To illustrate the characteristics described above, we simulated a single data set (*N* = 10000) based on a correlated factor model with three correlated facets. Each factor is measured with three indicators. In the population model, the standardized factor loadings of all facets were $\lambda_{T1j}$ = .6,$\lambda_{T2j}=$ .7, and $\lambda_{T3j}$ = .8. The correlations between the factors were set to $\varphi_{T1, T2}$ = .3, $\varphi_{T1, T3}$ = .3, and $\varphi_{T2, T3}$ = .7. The residual variance of each indicator was computed as ${Var(\varepsilon}_{i})=1-{\lambda^{2}}_{i}$. The variance of the latent variables was set to 1.

We fitted (1) a correlated factor model, as well as (2) a constraint and unconstraint latent regression model with *T*_1_ as reference facet, (3) a constraint and unconstrained BF*_S_*_–1_ model with *T*_1_ as reference facet, and (4) a constraint BF*_S_*_–1_ model with *T*_2_ and *T*_3_. All models are identified by fixing the factor loading of the first indicator to 1 and the factor means to zero. In the LR-UNC, the loadings of the first indicators of the non-reference factors on the reference factor were also fixed to zero (Chen et al., 2006; Yung et al., 1999). The syntaxes are provided online: https://osf.io/sq4zd/

**Results**

The correlated factor model, the LR-CON, and the BF*_S_*_–1_-CON models showed equivalent model fit (see Table S1.2). Importantly, they showed the same fit irrespective of which facet is used as reference facet. However, while the fit of the BF*_S_*_–1_-UNC and the LR-UNC was the same, both models showed fit slightly different from the fit of the constraint models. The differences are expected as the BF*_S_*_–1_-UNC and the LR-UNC are less restrictive due to the missing proportionality constraint (i.e., both models estimate more free parameters).

The estimated parameters show the expected pattern (see Table S1.3). The standardized and unstandardized factor loadings and the factor variance of the reference facet are the same in all models that use $T_{1}$ as the reference facet – irrespective of whether the constraint or unconstraint model is employed. In our simulated data set, the differences in the estimated parameters of the indicators of the non-reference facet between the constrained and unconstrained models were minimal. More considerable differences can be expected in empirical applications with more heterogeneous indicators. The residual variances of the non-reference facets in the latent regression model are equivalent to the *S* factor variances in the constrained BF*_S_*_–1_-CON models. The parameter equivalence illustrates that the reference facet has the same meaning in all models that use the same facet as the reference.

| **Table S1.1**  Variances and Covariances implied by the latent regression model, the unconstraint BF*_S_*_–1,_ and the constraint BF*_S_*_–1_ model | |
| --- | --- |
| Implied variance for an indicator *i* of the reference facet *j,* that is, ${Var(Y}_{ij})$ | |
| LR-CON | (5a)$\lambda_{Tij}^{2} \cdot{Var(T}_{j})+{Var(\varepsilon}_{ij})$ |
| LR-UNC | (5b) $\lambda_{Tij}^{2} \cdot{Var(T}_{j})+{Var(\varepsilon}_{ij})$ |
| BF*_S_*_–1_-UNC | (5c) $\lambda_{Gij}^{2} \cdot{Var(G}_{j})+{Var(\varepsilon}_{ij})$ |
| BF*_S_*_–1_-CON | (5d) $\lambda_{Gij}^{2} \cdot{Var(G}_{j})+{Var(\varepsilon}_{ij})$ |
| Implied variance for an indicator *i* of an arbitrary non-reference facet *j´* in a model in which *j* denotes the reference facet, ${Var(Y}_{ij´})$ | |
| LR-CON | (6a) $\lambda_{Tij´}^{2}\cdot{\beta_{jj´}^{2} \cdot Var(T}_{j})+\lambda_{Tij´}^{2} {\cdot Var(S}_{j´}) {+ Var(\varepsilon}_{ij´})$ |
| LR-UNC | (6a) $(\beta_{jij´}^{D}+ \lambda_{Tij´}\cdot{\beta_{jj´})^{2}\cdot Var(T}_{j})+\lambda_{Tij´}^{2} {\cdot Var(S}_{j´}) {+ Var(\varepsilon}_{ij´})$ |
| BF*_S_*_–1_-UNC | (6c) $\lambda_{Gij´}^{2} \cdot{Var(G}_{j})+\lambda_{Sij´}^{2} {\cdot Var(S}_{j´}) {+ Var(\varepsilon}_{ij´})$ |
| BF*_S_*_–1_-CON | (6d) $\lambda_{Sij´}^{2}\cdot\beta_{jj´}^{2} \cdot{Var(G}_{j})+\lambda_{Sij´}^{2} {\cdot Var(S}_{j´}) {+ Var(\varepsilon}_{ij´})$ |
| Implied covariance between two reference indicators *i* and *i´* of the reference facet *j*, ${Cov(Y}_{ij}, Y_{i´j})$ | |
| LR-CON | (7a) $\lambda_{Tij}\cdot\lambda_{Ti´j}\cdot{Var(T}_{j})$ |
| LR-UNC | (7b) $\lambda_{Tij}\cdot\lambda_{Ti´j}\cdot{Var(T}_{j})$ |
| BF*_S_*_–1_-UNC | (7c) $\lambda_{Gij}\cdot\lambda_{Gi´j}\cdot{Var(G}_{j})$ |
| BF*_S_*_–1_-CON | (7d) $\lambda_{Gij}\cdot\lambda_{Gi´j}\cdot{Var(G}_{j})$ |
| Implied covariance one indicator *i* of reference facet *j* and one non-reference indicator *i* of the non-reference facet *j´,* ${Cov(Y}_{ij}, Y_{ij´})$ | |
| LR-CON | (8a) $\lambda_{Tij}\cdot{\beta_{jj'}\cdot\lambda_{Tij^{'}} \cdot Var(T}_{j})$ |
| LR-UNC | (8b) $\lambda_{Tij}\cdot({\beta_{jj'}\cdot\lambda_{Tij^{'}}+ \beta_{jij´}^{D}) \cdot Var(T}_{j})$ |
| BF*_S_*_–1_-UNC | (8c) $\lambda_{Gij} \cdot\lambda_{Gij´} \cdot{Var(G}_{j})$ |
| BF*_S_*_–1_-CON | (8d) $\lambda_{Gij} \cdot\beta_{jj´}{\cdot\lambda}_{Sij´} \cdot{Var(G}_{j})$ |
| Implied covariance between two indicators *i* and *i´* of the same non-reference facet *j´*, ${Cov(Y}_{ij´}, Y_{i´j´})$ | |
| LR-CON | (9a) $\beta_{jj´} \cdot\lambda_{Tij´} \cdot\beta_{jj´}\cdot\lambda_{Ti´j´} {\cdot Var(T}_{j})+ \lambda_{Tij´}\cdot\lambda_{Ti´j´}{\cdot Var(S}_{j´})$ |
| LR-UNC | (9b) ${(\beta}_{jj´}\cdot\lambda_{Tij´}+ \beta_{jij´}^{D}) \cdot({\beta_{jj´}\cdot\lambda_{Ti´j´}+ \beta_{ji´j´}^{D}) \cdot Var(T}_{j})+ \lambda_{Tij´}\cdot\lambda_{Ti´j´}{\cdot Var(S}_{j´})$ |
| BF*_S_*_–1_-UNC | (9c)$\lambda_{Gij´} \cdot\lambda_{Gi´j´} {\cdot Var(G}_{j})+ \lambda_{Sij´}\cdot\lambda_{Si´j´}{\cdot Var(S}_{j´})$ |
| BF*_S_*_–1_-CON | (9d) $\beta_{jj´}{\cdot\lambda}_{Sij´} \cdot\beta_{jj´}\cdot\lambda_{Si´j´} {\cdot Var(G}_{j})+ \lambda_{Sij´}\cdot\lambda_{Si´j´}{\cdot Var(S}_{j´})$ |
| Implied covariance between two indicators *i* of two different non-reference facets *j´* and *j´´*, ${Cov(Y}_{ij´}, Y_{ij´´})$ | |
| LR-CON | (10a) $\beta_{jj´}{{\cdot\lambda_{Tij´} \cdot\beta_{jj´´} \cdot\lambda}_{Tij´´} \cdot Var(T}_{j})+\lambda_{Tij´} {\cdot\lambda}_{Tij´´}{\cdot Cov(S}_{j´}, S_{j´´})$ |
| LR-UNCON | (10b) ${(\beta}_{jj´}\cdot\lambda_{Tij´}+ \beta_{jij´}^{D})\cdot({\beta_{jj´´}\cdot\lambda_{Tij´´}+ \beta_{jij´´}^{D}) \cdot Var(T}_{j})+\lambda_{Tij´} {\cdot\lambda}_{Tij´´}{\cdot Cov(S}_{j´}, S_{j´´})$ |
| BF*_S_*_–1_-UNCON | (10c) $\lambda_{Gij´} \cdot{\lambda_{Gij"} \cdot Var(G}_{j})+\lambda_{Sij´} {\cdot\lambda}_{Sij´´}{\cdot Cov(S}_{j´}, S_{j´´})$ |
| BF*_S_*_–1_-CON | (10d) $\beta_{jj´} \cdot\lambda_{Sij´} \cdot\beta_{jj"} \cdot{\lambda_{Sij"} \cdot Var(G}_{1})+\lambda_{Sij´} {\cdot\lambda}_{Sij´´}{\cdot Cov(S}_{j´}, S_{j´´})$ |

| **Table S1.2**  Model-fit indices | | | | | | |
| --- | --- | --- | --- | --- | --- | --- |
| Model | #p | χ² (df) | *p* | RMSEA | SRMR | CFI |
| Correlated Factor Model | 30 | 25.821 (24) | .362 | .003 | .005 | 1 |
| LR-CON, *T*_1_ as Reference | 30 | 25.821 (24) | .362 | .003 | .005 | 1 |
| BF*_S_*_–1_-UNC, *T*_1_ as Reference | 34 | 24.241 (20) | .232 | .005 | .004 | 1 |
| BF*_S_*_–1_ -CON, *T*_1_ as Reference | 30 | 25.821 (24) | .362 | .003 | .005 | 1 |
| BF*_S_*_–1_-CON, *T*_2_ as Reference | 30 | 25.821 (24) | .362 | .003 | .005 | 1 |
| BF*_S_*_–1_-CON, *T*_3_ as Reference | 30 | 25.821 (24) | .362 | .003 | .005 | 1 |
| LR-UNC, T1 as Reference | 34 | 24.241 (20) | .232 | .005 | .004 | 1 |
| *Note.* #p= number of free parameters. BF*_S_*_–1_ = Bifactor- (*S−*1) model | | | | | | |

| **Table S1.3**  Parameters of the Correlated Factor Model, the Latent Regression Models with T1 as Reference, and Bifactor-(S-1) Models with Different Reference Facets | | | | | | | | | | | | | | | | | | | |
| --- | --- | --- | --- | --- | --- | --- | --- | --- | --- | --- | --- | --- | --- | --- | --- | --- | --- | --- | --- |
| Factor | Item | CFM |  | LR-CON Ref: T1 |  | BF*_S_*_–1_ -UCO  Ref: T1 | |  | BF*_S_*_–1_ -CON  Ref: T1 | |  | BF*_S_*_–1_ -CON  Ref: T2 | |  | BF*_S_*_–1_ -CON  Ref: T3 | |  | LR-UCO  Ref: T1 | |
|  |  | $\lambda_{T}$($\lambda_{T,STD}$) |  | $\lambda_{T}$($\lambda_{T,STD}$) |  | $\lambda_{G1}$($\lambda_{G1,STD}$) | $\lambda_{S}$($\lambda_{S,STD}$) |  | $\lambda_{G1}$($\lambda_{G1,STD}$) | $\lambda_{S}$($\lambda_{S,STD}$) |  | $\lambda_{G2}$($\lambda_{G2,STD}$) | $\lambda_{S}$($\lambda_{S,STD}$) |  | $\lambda_{G3}$($\lambda_{G3, STD}$) | $\lambda_{S}$($\lambda_{S,STD}$) |  | $\lambda_{T1}$($\lambda_{T1,STD}$) | $\lambda_{T}$($\lambda_{T,STD}$) |
| T1 | Y11 | 1.00 (.61) |  | 1.00 (.61) |  | 1.00 (.61) |  |  | 1.00 (.61) |  |  | 0.29 (.17) | 1.00 (.58) |  | 0.31 (.18) | 1.00 (.58) |  | 1.00 (.61) |  |
|  | Y21 | 1.17 (.71) |  | 1.17 (.71) |  | 1.17 (.71) |  |  | 1.17 (.71) |  |  | 0.34 (.20) | 1.17 (.68) |  | 0.36 (.21) | 1.17 (.68) |  | 1.17 (.71) |  |
|  | Y31 | 1.32 (.79) |  | 1.32 (.79) |  | 1.32 (.79) |  |  | 1.32 (.79) |  |  | 0.38 (.23) | 1.32 (.76) |  | 0.41 (.24) | 1.32 (.76) |  | 1.32 (.79) |  |
| T2 | Y12 | 1.00 (.60) |  | 1.00 (.60) |  | 0.28 (.17) | 1.00 (.57) |  | 0.29 (.17) | 1.00 (.57) |  | 1.00 (.60) |  |  | 0.72 (.41) | 1.00 (.43) |  | 0 | 1.00 (.60) |
|  | Y22 | 1.14 (.69) |  | 1.14 (.69) |  | 0.32 (.19) | 1.14 (.66) |  | 0.33 (.20) | 1.14 (.66) |  | 1.14 (.69) |  |  | 0.81 (.48) | 1.14 (.50) |  | -0.01 (-0.00) | 1.14 (.69) |
|  | Y32 | 1.32 (.80) |  | 1.32 (.80) |  | 0.38 (.23) | 1.32 (.77) |  | 0.38 (.23) | 1.32 (.77) |  | 1.32 (.80) |  |  | 0.95 (.55) | 1.32 (.58) |  | 0.01 (0.01) | 1.32 (.80) |
| T3 | Y13 | 1.00 (.59) |  | 1.00 (.59) |  | 0.28 (.17) | 1.00 (.56) |  | 0.29 (.17) | 1.00 (.56) |  | 0.66 (.40) | 1.00 (.43) |  | 1.00 (.59) |  |  | 0 | 1.00 (.59) |
|  | Y23 | 1.20 (.70) |  | 1.20 (.70) |  | 0.35 (.21) | 1.19 (.67) |  | 0.34 (.21) | 1.20 (.67) |  | 0.79 (.48) | 1.20 (.51) |  | 1.20 (.70) |  |  | 0.02 (0.01) | 1.20 (.70) |
|  | Y33 | 1.35 (.80) |  | 1.35 (.80) |  | 0.38 (.24) | 1.35 (.76) |  | 0.39 (.24) | 1.35 (.76) |  | 0.90 (.55) | 1.35 (.58) |  | 1.35 (.80) |  |  | 0.01 (0.01) | 1.35 (.80) |
| Factor Variances | | | | | | | | | | | | | | | | |  |  |  |
| T1 |  | 0.37 |  | 0.37 |  | 0.37 |  |  | 0.37 |  |  | 0.34^R^ |  |  | 0.34^R^ |  |  | 0.37 |  |
| T2 |  | 0.37 |  | 0.34^R^ |  | 0.34^R^ |  |  | 0.34^R^ |  |  | 0.37 |  |  | 0.19^R^ |  |  | 0.34^R^ |  |
| T3 |  | 0.34 |  | 0.31^R^ |  | 0.31^R^ |  |  | 0.31^R^ |  |  | 0.18^R^ |  |  | 0.34 |  |  | 0.31^R^ |  |
| Covariances | | | | | | | | | | | | | | | | |  |  |  |
| T1, T2 |  | 0.11 (.29) |  | 0.29 (.29) ^B^ |  | 0 |  |  | 0 |  |  | 0 |  |  | 0.03 (.12) ^P^ |  |  | .28 (.28) ^B^ |  |
| T1, T3 |  | 0.11 (.30) |  | 0.29 (.30) ^B^ |  | 0 |  |  | 0 |  |  | 0.04 (.14 ) ^P^ |  |  | 0 |  |  | .28 (.29) ^B^ |  |
| T2, T3 |  | 0.24 (.69) |  | 0.21 (.66) ^P^ |  | 0.22 (.66)^P^ |  |  | 0.21 (.66) ^P^ |  |  | 0 |  |  | 0 |  |  | 0.22 (.66)^P^ |  |
| *Note.*  CFM = Correlated-Factor Model. BF*_S_*_–1_ -CON = constraint BF*_S_*_–1_  model. BF*_S_*_–1_ -UCO = unconstraint BF*_S_*_–1_  model. LR -CON = constraint latent regression model. LR -UCO = unconstraint latent regression model. $\lambda_{Gj,STD}$ = Unstandardized and standardized general factor loading, the superscript *j* indicates the facet used as reference. $\lambda_{S}$ and $\lambda_{S,STD}$= Unstandardized and standardized *S* factor loadings. ^R^ = Residual variance controlled for the reference facet.  ^P^ = Partial correlation once the reference facet was considered.  ^B^ = regression weights. | | | | | | | | | | | | | | | | | | | |

**Figure S1.1**

Relation between Different Latent Regression and Bifactor-(S-1) Models (Figure adopted from Yung et al. 1999)


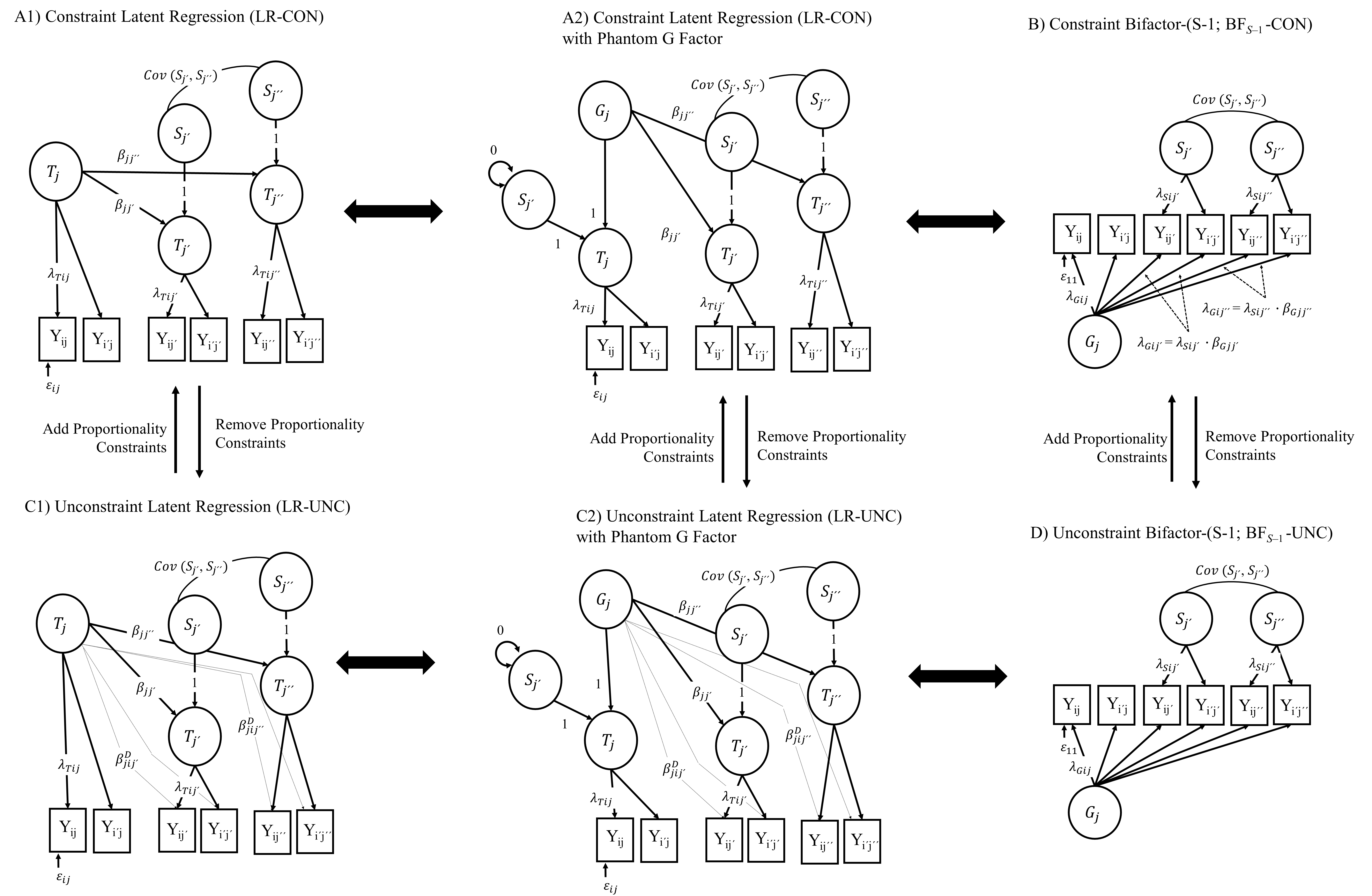


**Supplemental Material 2**

This supplemental material illustrates the effect of a collapsing factor on the meaning of the general factor in a second empirical data set. We use data from *N* = 2473 (*M_age_* = 44.2, 63.9% women) individuals assessed with the Beck Depression Inventory-II in a randomized controlled trial exploring the efficacy of internet-based intervention for depressive symptoms (Zagorscak et al., 2018, trial-registry: ACTRN12616001521415). We fit the BDI-II measurement model developed by Vanheule et al. (2008).

This model is based on Buckley et al. (2001) and includes only 15 of the original 21 BDI-II items. The model comprises three factors representing affective complaints (3 items), cognitive complaints (6 items), and somatic complaints (6 items). Two questions of the Patient Health Questionnaire - Stress Module (PHQ-S; Gräfe et al., 2004) assessing *worries about health* and *work- or school-related stress* served as external criteria.

The illustrative example differs from the empirical illustration in the manuscript. The items employed in the manuscript are conceptually interchangeable and approach the ideal of reflective indicators. Thus, they are valid and reliable (e.g., show very high standardized factor loadings on their facet factors) and homogenous (e.g., show similar high factor loadings). The indicators used in this additional example lack these advantageous properties. For example, the affective complaints factor is measured with items assessing loss of pleasure, loss of interest, and crying. Thus, the item crying stands out and measures something different than anhedonia. Therefore, instead of treating all items as indicators of the same construct (as intended by the employed measurement model), it would probably make sense to represent each symptom as a separate latent variable. We ignore this and show that the effect of collapsing factors is the same irrespective of whether reference indicators are homogenous or heterogeneous. The syntaxes are provided online: https://osf.io/sq4zd/

**Results.** The correlated factor model showed an acceptable fit, and the factor loadings showed considerable heterogeneity within each facet (see Table S2.1). This heterogeneity is expectable as items assigned to represent the same facets assess somewhat distinct and heterogenous symptoms of depression.

Next, we added a general factor. The fit of the BF_SYM_ model was superior to the model fit of the correlated factor model. However, the BF_SYM_ model showed anomalous results. The specific factor measuring affective complaints showed an indication of factor collapse. The specific factor was difficult to interpret, as the crying item changed sign differently compared to the other items (standardized specific factor loadings: λ_BDI4_ = .19, *SE* = .05, *p* < .001, λ_BDI10_ = -.40, *SE* = .16, *p* = .015, λ_BDI12_ = .24, *SE* = .07, *p* = .001). Moreover*,* the standardized factor loadings of BDI-II item 4 and BDI-II item 12 were small, albeit statistically significant. These low standardized loadings are mirrored by a small specific affective complaints factor variance (0.04, *SE* = 0.02, *p* = .074). These results indicate that the BF_SYM_ model became an empirical BF*_S_*_-1_ model with the affective complaints factor as a reference facet. The general factor represents the latent variable underlying the indicators of the collapsing factor.

As is typical in empirical applications, we removed the collapsing specific factor. The resulting BF*_S_*_-1_ model with affective complaints as a reference facet fits the data well (see Table S2.1). The partial correlation between the specific somatic complaints and specific cognitive complaints factor was close to zero (-.05), indicating that specific factors have nothing in common once the affective factor is partialed out.

The standardized and unstandardized first-order loadings of the three indicators assessing affective complaints in the correlated factor model were very similar to the general factor loadings of these indicators in the BF*_S_*_-1_ and BF_SYM_ model. Thus, the indicators show the same relationship to the latent variable in all three models.

The correlations between the factor scores and the correlations between the latent variables and the two covariates provide further empirical support that the general factor represents the latent variable underlying the collapsing factor's indicators. The factor scores of the general factor in the BF_SYM_ model correlated almost perfectly with (1) factor scores of the BF*_S_*_-1_ model with affective complaints as reference domain (.997), and with (2) factor scores of the affective complaints factor in the model with correlated factors (.995). Moreover, the correlations between the stress items and general factor in the BF_SYM_ model (see Table S2.2) with the collapsing *S* factor (.23 and .12) were the same as the correlations between the stress items and (1) the affective complaints reference facet of the BF*_S_*_-1_ model (.23 and .12, respectively), and (2) the affective complaints factor in the model with correlated factors (.23 and .12, respectively). The results are consistent with the arguments presented in the manuscript and indicate that researchers could model the latent variable that the BF_SYM_ model considers the "general factor" using only the three indicators of the collapsing factor.

| **Table S2.1**  Estimated model parameters | | | | | | | | | |
| --- | --- | --- | --- | --- | --- | --- | --- | --- | --- |
|  |  |  |  |  |  | |  | BF*_S_*_-1_ | |
| Factor | Item |  | CFM |  | BF_SYM_ | |  | AFF*_S-_*_COG,_ *_S-_*_SOM_ | |
|  |  |  | λ |  | λ*_G_* | λ_S_ |  | λ_G-AFF_ | λ_S_ |
| AFF | BDI-II 4 |  | 1.00 (.76) |  | 1.00 (.75) | 1.00 (.19) |  | 1.00 (.75) |  |
|  | BDI-II 10 |  | 0.65 (.49) |  | 0.71 (.53) | -2.11 (-.40) |  | 0.65 (.49) |  |
|  | BDI-II 12 |  | 0.98 (.74) |  | 0.98 (.73) | 1.28 (.24) |  | 0.98 (.74) |  |
| COG | BDI-II 2 |  | 1.00 (.66) |  | 0.71 (.53) | 1.00 (.30) |  | 0.70 (.53) | 1.00 (.30) |
|  | BDI-II 3 |  | 1.01 (.66) |  | 0.56 (.42) | 1.78 (.54) |  | 0.57 (.43) | 1.79 (.54) |
|  | BDI-II 6 |  | 0.82 (.54) |  | 0.48 (.36) | 1.33 (.41) |  | 0.49 (.37) | 1.33 (.40) |
|  | BDI-II 8 |  | 1.00 (.66) |  | 0.58 (.43) | 1.67 (.51) |  | 0.58 (.44) | 1.66 (.50) |
|  | BDI-II 9 |  | 0.80 (.53) |  | 0.53 (.40) | 1.01 (.31) |  | 0.53 (.40) | 1.01 (.30) |
|  | BDI-II 4 |  | 1.16 (.76) |  | 0.64 (.48) | 2.17 (.66) |  | 0.64 (.48) | 2.17 (.65) |
| SOM | BDI-II 6 |  | 1.00 (.36) |  | 0.38 (.29) | 1.00 (.27) |  | 0.39 (.30) | 1.00 (.26) |
|  | BDI-II 17 |  | 1.27 (.47) |  | 0.54 (.40) | 0.72 (.20) |  | 0.54 (.41) | 0.66 (.17) |
|  | BDI-II 18 |  | 1.40 (.51) |  | 0.60 (.45) | 0.65 (.18) |  | 0.60 (.46) | 0.59 (.15) |
|  | BDI-II 19 |  | 1.73 (.63) |  | 0.72 (.54) | 1.06 (.29) |  | 0.73 (.55) | 1.00 (.26) |
|  | BDI-II 20 |  | 1.76 (.64) |  | 0.69 (.51) | 2.12 (.58) |  | 0.70 (.53) | 2.37 (.61) |
|  | BDI-II 21 |  | 1.19 (.44) |  | 0.53 (.40) | 0.39 (.11) |  | 0.53 (.40) | 0.40 (.10) |
| Variances (Standard Deviations) | | | |  |  |  |  |  |  |
|  | G |  |  |  | 0.56 (1) | |  |  |  |
|  | AFF |  | 0.57 (1) |  | 0.04 (1) | |  | 0.57 (1) | |
|  | COG |  | 0.43 (1) |  | 0.09 (1) | |  | 0.09 (1) | |
|  | SOM |  | 0.13 (1) |  | 0.08 (1) | |  | 0.07 (1) | |
| Covariances (Correlations) | | |  |  |  |  |  |  |  |
|  | AFF, COG |  | 0.34 (.68) |  | ^F0^ |  |  | ^F0^ |  |
|  | AFF, SOM |  | 0.24 (.86) |  | ^F0^ |  |  | ^F0^ |  |
|  | COG, SOM |  | 0.14 (.58) |  | ^F0^ |  |  | -0.004 (-.05) | |
| Model Fit | | | | | | | | | |
|  | *χ*² |  | 647.817 |  | 451.433 |  |  | 496.252 | |
|  | *df* |  | 87 |  | 75 |  |  | 77 | |
|  | *p* |  | < .001 |  | < .001 |  |  | < .001 | |
|  | RMSEA [90% CI] |  | .051  [.047, .055] |  | .045 [.041, .059] |  |  | .047 [.043, .051] | |
|  | SRMR |  | .035 |  | .029 |  |  | .031 | |
|  | CFI |  | .959 |  | .973 |  |  | .970 | |
| *Note*. AFF = Affective complaints. COG = Cognitive complaints. SOM = Somatic complaints. λ = factor loading. λ_G_ = loading on the general factor, standardized loading in brackets. λ_S_ = loading on the specific factor, standardized loadings in brackets. Conceptual similar parameters are highlighted in colors. | | | | | | | | | |

| **Table S2.2**  Correlations (covariances) between the latent variables and the PHQ-S items. | | | | | | | | | | |
| --- | --- | --- | --- | --- | --- | --- | --- | --- | --- | --- |
| Model |  | Worries about health | | | |  | Work- or school-related stress | | | |
|  |  | G | AFF | COG | SOM |  | G | AFF | COG | SOM |
| CFA |  |  | 0.23 (.30) | 0.12 (.17) | 0.16 (.43) |  |  | 0.12 (.16) | 0.14 (.19) | 0.12 (.33) |
| BF*_S_*_YM_ |  | 0.23 (.30) | -0.002 (-.01) | -0.01 (-.05) | 0.11 (.33) |  | 0.12 (.16) | -0.01 (-.05) | 0.04 (.12) | 0.12 (.38) |
| BF*_S_*_-1,_  AFF*_S-_*_COG,_ *_S-_*_SOM_ |  |  | 0.23 (.30) | -0.01 (-.05) | 0.10 (.33) |  |  | 0.12 (.15) | 0.04 (.12) | 0.12 (.38) |
| *Note*. AFF = Affective complaints. COG = Cognitive complaints. SOM = Somatic complaints. Conceptual similar associations are highlighted in the same color. | | | | | | | | | | |

**References**

Buckley, T. C., Parker, J. D., & Heggie, J. (2001). A psychometric evaluation of the BDI-II in treatment-seeking substance abusers. *Journal of Substance Abuse Treatment*, *20*(3), 197–204. https://doi.org/10.1016/S0740-5472(00)00169-0

Burns, G. L., Geiser, C., Servera, M., Becker, S. P., & Beauchaine, T. P. (2020). Promises and pitfalls of latent variable approaches to understanding psychopathology: Reply to Burke and Johnston, Eid, Junghänel and Colleagues, and Willoughby. *Journal of Abnormal Child Psychology*, *48*(7), 917–922. https://doi.org/10.1007/s10802-020-00656-1

Caspi, A., Houts, R. M., Belsky, D. W., Goldman-Mellor, S. J., Harrington, H., Israel, S., Meier, M. H., Ramrakha, S., Shalev, I., Poulton, R., & Moffitt, T. E. (2014). The p Factor: One general psychopathology factor in the structure of psychiatric disorders? *Clinical psychological science*, *2*(2), 119–137. https://doi.org/10.1177/2167702613497473

Chen, F. F., West, S., & Sousa, K. (2006). A Comparison of Bifactor and Second-Order Models of Quality of Life. *Multivariate Behavioral Research*, *41*(2), 189–225. https://doi.org/10.1207/s15327906mbr4102_5

Eid, M., Geiser, C., Koch, T., & Heene, M. (2017). Anomalous results in G-factor models: Explanations and alternatives. *Psychological Methods*, *22*(3), 541–562. https://doi.org/10.1037/met0000083

Geiser, C., Eid, M., & Nussbeck, F. W. (2008). On the meaning of the latent variables in the CT-C(M-1) model: A comment on Maydeu-Olivares and Coffman (2006). *Psychological Methods*, *13*(1), 49–57. https://doi.org/10.1037/1082-989X.13.1.49

Geiser, C., Eid, M., West, S. G., Lischetzke, T., & Nussbeck, F. W. (2012). A comparison of method effects in two confirmatory factor models for structurally different methods. *Structural Equation Modeling: A Multidisciplinary Journal*, *19*(3), 409–436. https://doi.org/10.1080/10705511.2012.687658

Gignac, G. E. (2016). The higher-order model imposes a proportionality constraint: That is why the bifactor model tends to fit better. *Intelligence*, *55*, 57–68. https://doi.org/10.1016/j.intell.2016.01.006

Gräfe, K., Zipfel, S., Herzog, W., & Löwe, B. (2004). Screening psychischer Störungen mit dem “Gesundheitsfragebogen für Patienten (PHQ-D)“. *Diagnostica*, *50*(4), 171–181.

Heinrich, M., Zagorscak, P., Eid, M., & Knaevelsrud, C. (2020). Giving G a meaning: An application of the bifactor-(S-1) approach to realize a more symptom-oriented modeling of the Beck Depression Inventory–II. *Assessment*, *27*(7). https://doi.org/10.1177/1073191118803738

Koch, T., Holtmann, J., Bohn, J., & Eid, M. (2018). Explaining general and specific factors in longitudinal, multimethod, and bifactor models: Some caveats and recommendations. *Psychological Methods*, *23*(3), 505–523. https://doi.org/10.1037/met0000146

Schmiedek, F., & Li, S.-C. (2004). Toward an alternative representation for disentangling age-associated differences in general and specific cognitive abilities. *Psychology and Aging*, *19*(1), 40–56. https://doi.org/10.1037/0882-7974.19.1.40

Vanheule, S., Desmet, M., Groenvynck, H., Rosseel, Y., & Fontaine, J. (2008). The factor structure of the Beck Depression Inventory–II: An evaluation. *Assessment*, *15*(2), 177–187. https://doi.org/10.1177/1073191107311261

Yung, Y.-F., Thissen, D., & McLeod, L. D. (1999). On the relationship between the higher-order factor model and the hierarchical factor model. *Psychometrika*, *64*(2), 113–128. https://doi.org/10.1007/BF02294531

Zagorscak, P., Heinrich, M., Sommer, D., Wagner, B., & Knaevelsrud, C. (2018). Benefits of individualized feedback in internet-based interventions for depression: A randomized controlled trial. *Psychotherapy and Psychosomatics*, *87*(1), 32–45. https://doi.org/10.1159/000481515
